# The speckle-type POZ protein (SPOP) inhibits breast cancer malignancy by destabilizing TWIST1

**DOI:** 10.1038/s41420-022-01182-3

**Published:** 2022-09-17

**Authors:** Chunli Wei, Yun Liu, Xiaoyan Liu, Jingliang Cheng, Jiewen Fu, Xiuli Xiao, Robb E. Moses, Xiaotao Li, Junjiang Fu

**Affiliations:** 1 Key Laboratory of Epigenetics and Oncology, the Research Center for Preclinical Medicine, Southwest Medical University, Luzhou, 646000 Sichuan China; 2 Shanghai Key Laboratory of Regulatory Biology, Institute of Biomedical Sciences, School of Life Sciences, East China Normal University, Shanghai, 200241 China; 3 Department of pathology, Southwest Medical University, Luzhou, 646000 Sichuan China; 4 Department of Molecular and Cellular Biology, Dan L. Duncan Cancer Center, Baylor College of Medicine, One Baylor Plaza, Houston, TX 77030 USA; 5 School of Arts and Sciences, New York University-Shanghai, 1555 Century Avenue Pudong, Shanghai, 200135 China

**Keywords:** Gene regulation, Tumour biomarkers

## Abstract

Epithelial-mesenchymal transition (EMT) inducing transcription factor TWIST1 plays a vital role in cancer metastasis. How the tumor-suppressive E3 ligase, speckle-type POZ protein (SPOP), regulates TWIST1 in breast cancer remains unknown. In this study, we report that SPOP physically interacts with, ubiquitinates, and destabilizes TWIST1. SPOP promotes K63-and K48-linked ubiquitination of TWIST1, predominantly at K73, thereby suppressing cancer cell migration and invasion. Silencing SPOP significantly enhances EMT, which accelerates breast cancer cell migration and invasiveness in vitro and lung metastasis in vivo. Clinically, SPOP is negatively correlated with the levels of TWIST1 in highly invasive breast carcinomas. Reduced SPOP expression, along with elevated TWIST1 levels, is associated with poor prognosis in advanced breast cancer patients, particularly those with metastatic triple-negative breast cancer (TNBC). Taken together, we have disclosed a new mechanism linking SPOP to TWIST1 degradation. Thus SPOP may serve as a prognostic marker and a potential therapeutic target for advanced TNBC patients.

## Introduction

The transcription factor TWIST1 is identified as a basic helix-loop-helix (bHLH) protein [1–3]. In mammals TWIST1 is developmentally expressed in mesoderm-derived embryonic tissues and in postnatal adult mesoderm-derived mesenchymal stem cells, functions as the main regulator for mesenchymal cell differentiation [4, 5]. Inactivating mutations in the *TWIST1* gene in human-cause the autosomal dominant inherited form of the disease, Saethre–Chotzen syndrome [6, 7]. In humans, TWIST1 plays vital oncogenic roles in various cancers including the generation of cancer cell stemness, drug resistance, epithelial-mesenchymal transition (EMT), and metastasis [8–10]. In the EMT processes, cells lose cell-cell adhesion and polarity, gaining invasive ability by down-regulation of epithelial markers such as E-cadherin, along with the acquisition of mesenchymal markers/phenotypes such as N-cadherin and Vimentin as hallmarks [11–13]. Thus EMT-associated transcription factors (EMT-TFs), such as TWIST1/2 and SNAIL1/2, can activate EMT and promote cancer metastasis [8, 14, 15]. Altered expression of TWIST1 has also been implied in the development of different cancers, including breast cancer [8, 9, 14].

The speckle-type POZ protein (SPOP) was first cloned as a novel antigen from a scleroderma patient in 1997 [16], and was identified as an E3 ubiquitin-protein ligase that mediates ubiquitination and degradation of targeted proteins through proteasomal pathways [17–19]. Cancer genome characterizations have identified missense variations in the *SPOP* gene in 11~13% of primary prostate cancer [20–22] and 6~8% of metastatic, castration-resistant prostate cancers (CRPC) [23]. Numerous studies indicate that SPOP suppresses tumor progress in many human malignancies such as prostate, lung, gastric, colon, and liver cancers [24, 25], but not well studied in breast cancers.

Breast cancer is the most common malignancy and the second cause of death in women worldwide [26–28]. Triple-negative breast cancer (TNBC) or advanced breast cancer is generally more aggressive with high risks of recurrences, metastasis, and death, having limited therapeutic approaches. However, a low mutation frequency of *SPOP* gene is found in breast cancer [29, 30]. SPOP functions for tumorigenesis in breast cancer are not yet known [31]. The relationship between SPOP and TWIST1 remains unknown. In this study, we elucidate a tumor-suppressive role of SPOP in advanced breast cancer by negatively controlling TWIST1 stability. The results indicate that as a mediator of TWIST1 destabilization, SPOP promotes metastasis of breast cancer cells and may serve as a potential target for personalized therapy and a clinical marker for disease prognosis.

## Materials and methods

### Plasmids, antibodies, and cell culture

Plasmid constructs for Flag-tagged TWIST1 (F-TWIST1), TRIM28 (F-TRIM28), HA-tagged ubiquitin (HA-Ub), antibodies for TWIST1, SPOP, E-cadherin, N-cadherin, Vimentin, Flag, HA, HSP70, β-actin, GST, and Tubulin, were as described previously [10, 31–33]. Full-length SPOP was purchased from Genechem (Genechem Co., Ltd., Shanghai, China) and sub-cloned into pcDNA5/FRT/TO vector (F-SPOP). GST-tagged C-terminal deletion mutant (GST-SPOPN) and N-terminal deletion mutant (GST-SPOPC) of SPOP were sub-cloned into pEGX4T1 vector (GST fusion vector). Different mutant forms of TWIST1 including TWIST1(K73R), TWIST1(K133R), TWIST1(K145R), TWIST1(K150R) through mutagenesis were constructed in pcDNA5/FRT/TO vector. Anti-Ubiquitin (linkage-specific K63) antibody (Abcam. ab179434), and anti-Ubiquitin (linkage-specific K48) antibody (Abcam. ab140601) were purchased from Abcam. Anti-SPOP antibody for Immunohistochemistry (IHC) (clone 9B7.1, Cat #: MABC565), Flag attached on M2 beads (F2426, ZEviewTM Red ANTI-FLAG M2 Affinity Gel, Sigma) were purchased from Sigma-Aldrich. The cancer cell lines BT549, MDA-MB-231, MDA-MB-468, MDA-MB-435, T47D, MCF7, 4T1 and HeLa, which were purchased from American Type Culture Collection (ATCC), were cultured in RPMI1640 or DMEM media (Thermo Fisher Scientific, USA) with 10% fetal bovine serum (FBS) in an incubator of 5% CO_2_ [10, 33].

### Western blotting, immunoprecipitation, immunohistochemistry and GST pull-down assays

Western blotting (WB), immunoprecipitation (IP) and immunohistochemistry (IHC) using cells and tissues were performed as described previously [10, 33]. GST, GST-SPOPN and GST-SPOPC were expressed in *E. coli* BL21 and then purified using glutathione-sepharose 4B breads (Amersham Pharmacia Biotech) [19]. The pull-down assays were done and the proteins on the beads were used for WB with indicated antibodies described [33]. We used the comprehensive image processing software Image J to perform gray-scale analysis of protein expression and quantify protein expression.

### Breast tumor sampling and protein isolation

The human breast invasive ductal carcinoma specimens and the matched normal adjacent tissues from Chinese women were collected with informed consent [10, 34]. Patients’ pathologic data were collected. A total of 58 breast invasive ductal carcinoma tissue samples and 26 adjacent normal tissues were obtained from the Affiliated Hospital of Southwest Medical University. Human studies were approved by the Southwest Medical University review board. Both cancer tissues and adjacent normal tissues were frozen immediately in liquid nitrogen. For extraction of proteins, the tissues were homogenized and lysed with ice-cold 1× EBC buffer with a protease inhibitor cocktail [35]. Western blotting assays were carried out with indicated antibodies.

### Assays for cycloheximide-based protein stability

Breast cancer cells T47D and BT549 were transfected with either shRNA control (Ctrl) or shRNA against SPOP (shSPOP66, shSPOP67) (Shanghai Genechem Co., Ltd., Shanghai, China) in 6 or 12 well plates. 2 days after cell transfection, cells were treated with 20 µg /ml of cycloheximide (CHX) (Sigma) at different times as indicated. Cells were harvested and lysed for western blotting. HEK293-TWIST1 inducible cell line and HEK293-TWIST1(K73R) inducible cell line were also used to do cycloheximide-based protein stability assay. Band intensities were semi-quantitatively analyzed by densitometry with Adobe Photoshop CS3 software. The degradation curves were plotted with the time period of CHX treatment as the X-axis and protein band intensities in logarithm as the Y-axis. The linear regression was drawn using Microsoft Excel software and the protein half-life was calculated [10].

### Ubiquitination assays

In vivo ubiquitination assays were conducted in BT549, MDA-MB-468 breast cancer cells, or HeLa cells co-transfected with plasmids expressing Flag-SPOP, Flag-TWIST1, or various mutants, and in the presence or absence of HA-ubiquitin (HA-Ub). Proteins were immune-precipitated with antibody where Flag was bound on M2 beads and applied for western blotting with antibodies against HA or Flag. In vivo ubiquitination assay for K63-linked, or K48-linked ubiquitinations, BT549 cells and MDA-MB-468 cells were transfected with a Flag-tagged SPOP construct along with MG132 treatment, and lysed cells for IP with TWIST1 antibody, followed by IB analysis with indicated antibodies.

### Cell migration and invasion assays

Real-time cell growth index, migration and invasion assays were described previously [33]. All assays were performed in a real time cell analyzer (xCELLigence RTCA DP, Roche, Germany) in triplet and repeated at least two times. Prior to the assays, breast cancer cell BT549 was transfected with either shRNA control (Ctrl) or shRNA against SPOP (shSPOP66, shSPOP67). To measure the index of cell growth, 100 μl of cell suspensions (5 × 10^4^ cells/ml) were seeded in each of the 16 well E-plate. CMI plates were applied for assays for cell migration and invasion with the lower chambers filled with chemotaxis inducer (10% FBS serum media) and upper chambers filled with 100 μl of cell suspensions (5 × 10^4^ cells/ml) with serum free medium. For cell invasion assay, the membrane of the CMI plate was coated with Matrigel (Catalogue #: 354277, BD Biosciences, USA) before the cells were seeded. Cell growth, migration and invasion status were monitored every 30 min. HEK293-TWIST1-inducible cell line and HEK293-TWIST1(K73R)-inducible cell line were also used for real-time cell growth index, migration and invasion assays with or without SPOP and ubiquitin overexpression. The relevant cell index value was recorded, and then the dose-response with variable slope formula in RTCA’s own analysis software was used to calculate and analyze the influence of cell invasion and migration. All experiments were repeated three times.

### Mouse xenograft model

Mouse xenograft assays were performed in accordance with the Declaration of Helsinki and national guidelines. For animal studies, we randomize and conduct blinded studies. Female nude mice were subcutaneously injected with cells in 120 μl of the serum free medium mixed with 80 μl growth factor-reduced Matrigel into fat pads at both sides of 4th mammary glands [31, 33]. The mice were randomly divided into three groups with six of them in each group (shRNA control, Vehicle; shRNA against SPOP: shSPOP66, shSPOP67). The body weight and tumor size were measured every three days. The volumes of the tumors were calculated by the following formula: tumor volume (mm^3^) = length (mm) × width (mm)^3^ × π/6 [36]. At endpoint, mice were euthanized as described previously [10]; tumors were dissected for subsequent analysis; the metastatic tumors appearing on the lung surface were carefully examined under a stereomicroscope.

### Protein purification and LC/MS mass spectrometry analysis

The protein purification from HEK293-TWIST1 inducible cell line was described previously [33]. The immune-precipitated proteins were resolved in SDS-PAGE and the Flag-TWIST1 protein band was excised from the gel and de-stained for in-gel digestion by trypsin. The tryptic peptides were extracted and subjected to Q-Exactive HF mass spectrometer (Thermo Fisher Scientific, Waltham, MA) for LC-MS/MS analysis. Tandem MS/MS data were acquired and LC-MS/MS results were subjected to protein identification for ubiquitin sites using software Maxquant (Version 1.5.8.3) with Andromeda search engine. The LC-MS/MS experiment was performed by Shanghai Bioprofile Technology Co., Ltd (Shanghai, China).

### Correlation and survival analysis

The Pearson correlation of SPOP and TWIST1 expression was conducted in normal breast tissues TCGA expression data through Gene Expression Profiling Interactive Analysis 2 (GEPIA 2) (http://gepia2.cancer-pku.cn/#correlation) [37–39]. Kaplan–Meier plotter was applied for overall survival (OS) in breast cancer patients with either low or high expression of TWIST1 and SPOP (https://kmplot.com/analysis/index.php?p=service&start=1). Analysis for subtypes for ER status, derived ER status from gene expression data, PR status, HER2 status, lymph node status, and grade were conducted [40]. Patients were categorized into low and high groups respectively, which were split by auto select best cutoff. The *p*‐values of survival analyses were computed using the log-rank test. *p*-value with less than 0.005 was considered a significant difference.

### Statistics analysis

ImageJ processing software was used to perform grayscale analysis of western blot results and semi-quantitate protein. Graph Pad Prism 9 was used to analyze relevant data in Mouse xenograft model. RTCA’s analysis software was used to analyze the influence of cell invasion and migration. Paired Student’s *t* test was applied to determine significant differences. *P*-values ≤ 0.05 were considered as significant differences. “*” indicates *p*-values ≤ 0.05, whereas “**” indicates *p*-values ≤ 0.01.

## Results

### Inverse correlation between the expressions of SPOP and TWIST1 in breast cancer cells

**Fig. 1:**
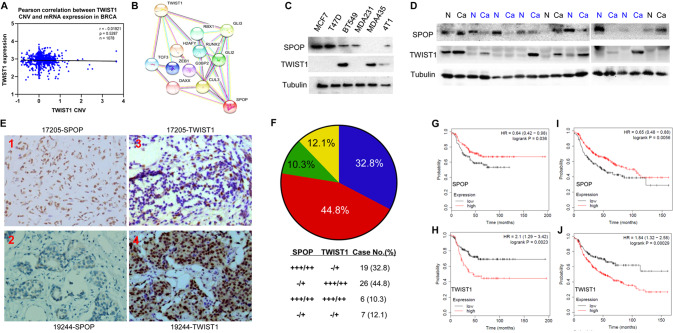
Expression of SPOP and TWIST1 is reversely correlated with invasive human breast carcinoma tissues, and associated with breast cancer patient survival among TNBC, positive lymph node status and high grade. **A** Analysis of TCGA breast cancer data. Pearson correlation between *TWIST1* mRNA expression and CNV in TCGA breast cancer. **B** Analysis of STRING Interaction Network to discover a potential link between TWIST1 and SPOP. **C** Protein expression levels for SPOP and TWIST1 in breast cancer cell lines. **D** Western blotting analysis for SPOP and TWIST1 protein expression in nine invasive ductal breast cancer tissues and their matched normal tissues. TWIST1 protein expression was inversely correlated with SPOP protein expression in the breast tumor tissues. **E** Representative Immunohistochemical (IHC) of indicated TWIST1 and SPOP in the tissues. Representative IHC for SPOP in tissue sample No. 17205 (1) and No. 19244 (2) respectively. Representative IHC for TWIST1 in tissue sample No. 17205 (3) and No. 19244 (4) respectively. **F** Reverse correlation of protein expression of SPOP and TWIST1 in breast invasive ductal carcinoma specimens. **G** High SPOP protein expression is positively correlated with patient survival of TNBC. **H** High TWIST1 protein expression is inversely correlated with patient survival of TNBC. **I** High SPOP protein expression is positively correlated with patient survival in both positive lymph node status and high grade. **J** High TWIST1 protein expression is inversely correlated with patient survival in patients with both positive lymph node status and high grades.

Given that our major focus was to address transcriptional and posttranslational regulation of *TWIST1* [10, 32–34, 41], we performed bioinformatics analysis to explore factors that may regulate these regulatory processes. To our surprise, amplified *TWIST1* copy number variation (CNV) did not correlate with increased expression of *TWIST1* in a TCGA breast cancer dataset (Fig. 1A) and a TNBC dataset (Supplementary Fig. S1A, B), suggesting a perturbed transcriptional regulation. Searching for interacting proteins that may regulate TWIST1 by STRING Interaction Network analysis, we discovered a potential link between TWIST1 and SPOP (Fig. 1B), a less-known E3 ligase in the breast cancer field [30]. Interestingly, initial testing with a panel of breast cancer cell lines revealed an inverse correlation between SPOP and TWIST1 (Fig. 1C).

To substantiate the correlation of SPOP and TWIST1 protein levels, we collected invasive ductal carcinoma tissues from Chinese breast cancer patients and adjacent normal tissues for analysis. By western blotting analysis, levels of TWIST1 in the majority of cancer tissues were significantly higher than in matched adjacent normal tissues (22 out of 26, Fig. 1D), whereas expression of SPOP in most cancer tissues was much lower than in matched adjacent tissues (21 of 26) (in Fig. 1D labeled blue “N”, Supplementary Fig. S2). Nearly all samples (regardless of tumor or non-tumor), particularly those labeled blue, displayed an inverse correlation between the expression of TWIST1 and SPOP (Fig. 1D, Supplementary Fig. S2). Furthermore, the inverse expression patterns of SPOP and TWIST1 were verified by immunohistochemistry analysis of breast cancer tissues (Fig. 1E, F). Among the 58 invasive ductal carcinoma specimens, nearly half of the samples (44.8%, 26/58) displayed a reduced SPOP expression along with increased TWIST1, whereas one-third (32.8%, 19/58) of tumors with higher SPOP had lower TWIST1 and 12.1% of tumors had no changes in either one (Fig. 1F). These data suggest that SPOP is downregulated in a significant number of invasive breast carcinomas with concomitant high expression of TWIST1, contributing to the development of advanced breast cancers.

### Association of lower SPOP and higher TWIST1 with poor survival in breast cancer patients

Kaplan–Meier survival analysis of breast patients (TNBC: *n* = 255; both positive lymph node status and high grade breast cancer: *n* = 369) showed that lower SPOP expression and higher TWIST1 expression were correlated with poor prognosis in TNBC patients [SPOP: HR 0.64 (0.42~0.98), *p*-value 0.036; TWIST1: HR 2.1 (1.29~3.42), *p*-value 0.0023] (Fig. 1G, H), as well as in patients with both positive lymph node invasion and high grades [SPOP: HR 0.65 (0.48~0.88), *p*-value 0.0056; TWIST1: HR 1.84 (1.32~2.58), *p*-value 0.00029] (Fig. 1I, J). Higher expression of SPOP positively correlated with longer survival in TNBC or high-grade breast cancer patients, whereas patients with increased levels of TWIST1 were associated with poor prognosis (Fig. 1G–J). These results substantiate that downregulation of SPOP is correlated with poor prognosis in breast cancer. Thus SPOP may function as a tumor suppressor to inhibit breast cancer progression to advanced stages, including triple negative, positive lymph node status and high grade. These data suggest that the roles of SPOP in regulation of breast cancer progression may be mediated, at least in part, by suppressing TWIST1.

### SPOP attenuates expression of TWIST1, altering related EMT markers

**Fig. 2:**
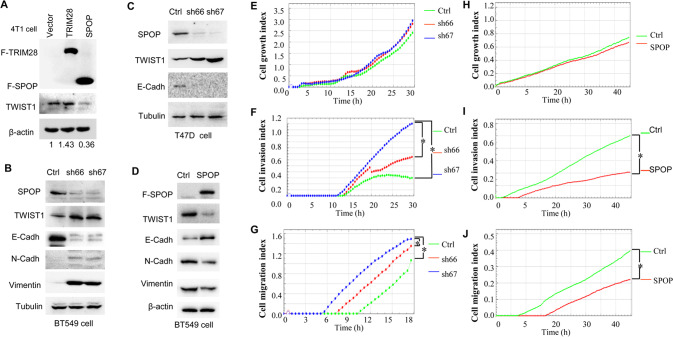
Knock-down of SPOP up-regulates TWIST1 and promotes breast cancer EMT, cell migration and invasion, whereas up-regulated SPOP suppresses EMT. **A** Over-expressed SPOP decreases TWIST1 protein levels. **B** Knock-down of SPOP up-regulates TWIST1 and facilitates expression of epithelial markers N-cadherin and Vimentin, and also downregulates mesenchymal marker E-cadherin in breast cancer cell BT549. Cells transfected with shCtrl or shSPOP (sh66 and sh67) vectors and protein lysates were collected at the indicated times for western blotting. **C** Knock-down of SPOP up-regulates TWIST1 and down-regulation of mesenchymal marker E-cadherin in breast cancer cell T47D. There is no detectable N-cadherin and Vimentin in T47D. **D** Over-expressed SPOP decreases TWIST1 protein levels and inhibits epithelial markers N-cadherin and Vimentin, and upregulates mesenchymal marker E-cadherin in breast cancer cell BT549. **E**–**G** Roles for cell index, cell migration and invasion by knock-down of SPOP in BT549 cell line. **H**–**J** Roles for cell index, cell migration and invasion by overexpression of SPOP in BT549 cell line. Real-time cell growth index, migration and invasion assays were performed in a real time cell analyzerr, *p*-value ≤ 0.05 was considered as a significant difference (*). N-cadh, N-cadherin. E-cadh E-cadherin.

We then asked whether overexpression of SPOP directly downregulates TWIST1 levels. Western blotting was performed to check TWIST1 expression in 4T1 cell transfected with SPOP, TRIM28 or an empty vector control. TRIM28 was used as a positive control due to its role in stabilization of TWIST1 protein [10]. Overexpression of SPOP diminished TWIST1 expression (Fig. 2A, lane 3), whereas exogenous expression of TRIM28 enhanced TWIST1 (Fig. 2A, lane 2), consistent with our previous reports for TRIM28 [10]. In contrast, silencing SPOP by two individual shRNA (sh66 and sh67) significantly up-regulated TWIST1 protein levels in BT549 cell (Fig. 2B). Yet, the mRNA levels of *TWIST1* were not changed in SPOP gain-of-function or loss-of-function experiments (Supplementary Fig. S3, and data not shown).

Down-regulation of cell-cell adhesion proteins, such as E-Cadherin, and up-regulation of plastic mesenchymal molecules, such as N-Cadherin and Vimentin are the hallmarks of TWIST1-mediated EMT. To determine whether SPOP could affect EMT, we silenced SPOP in both BT549 and T47D breast cancer lines and examined the effects on E-cadherin, Vimentin and N-cadherin by Western blotting. Successful depletion of SPOP led to reduction in E-Cadherin with concomitant increase in TWIST1, N-Cadherin and Vimentin (Fig. 2B, C). In contrast, overexpression of SPOP induced a reduction in TWIST1, N-Cadherin and Vimentin, but an increase in E-cadherin in BT549 (Fig. 2D). The observed loss-of-function in TWIST1 was not due to cytosolic and nuclear re-distribution of TWIST1 (Supplementary Fig. S4). Thus, our results support the notion that SPOP downregulates TWIST1 and inactivation of TWIST1 results in alteration of key EMT markers.

### Ablation of SPOP promotes breast cancer cell migration and invasion in vitro and in vivo

Since manipulation of SPOP significantly affected expression of TWIST1 and related EMT markers, we decided to dissect the role of SPOP in cell growth, cell migration and invasion. Knocking down SPOP in BT549 cells only slightly affected cell growth (Fig. 2E), but significantly promoted cell invasion (Fig. 2F) and migration (Fig. 2G). However, overexpressing SPOP in BT549 cells significantly attenuated cell invasion (Fig. 2I) and migration (Fig. 2J) with only minor effect on cell growth (Fig. 2H). Together with the observation that SPOP plays a vital role in regulating both TWIST1 and EMT markers, these data imply that SPOP plays an essential role in cell migration and invasion by regulating TWIST1 and EMT.

**Fig. 3:**
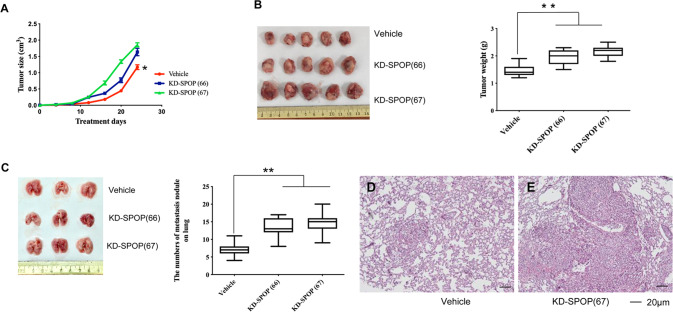
Knock-down of SPOP promotes tumor progress in vivo. **A** Knock-down of SPOP promotes tumor growth and tumor size. *P*-value ≤ 0.05 was considered as a significant difference (*). **B** Knock-down of SPOP promotes tumor growth in weight at the endpoint of scarified mouse. Left panel, representative tumor images; right panel, quantitative results. *P*-value ≤ 0.01 was considered as a significant difference (**). **C** Knock-down of SPOP promotes lung metastasis in vivo. Left panel, representative lung images; right panel, quantitative results of lung metastasis in vivo. *P*-value ≤ 0.01 was considered as a significant difference (**). **D** Representative lung tissue image of vehicle from **C**. **E** Representative lung tissue image of knock-down SPOP (clone 67) from **C**. KD-SPOP (66), knockdown SPOP in clone 66, KD-SPOP (67), knockdown SPOP in clone 67.

To evaluate the role of SPOP in growth and metastasis of breast cancer cells in vivo, we inoculated SPOP-knockdown (KD-SPOP (66, 67)) breast cancer cells or control (vehicle) cells into fat pads in the 4th mammary gland of nude mice and monitored xenograft tumor growth and metastasis. The results demonstrate that silencing SPOP indeed promoted tumor growth (Fig. 3A, B) with increased tumor volume and size. Importantly, orthotopic transplantation of SPOP knock-down breast cancer cells into the fat pad of the 4th mammary gland of nude mice resulted in enhanced lung metastasis compared to control cells treated with vehicle only (Fig. 3C–E). These data suggest that SPOP plays a vital role in breast cancer invasion and lung metastasis in animals as well.

### SPOP ubiquitinates and destabilizes TWIST1

**Fig. 4:**
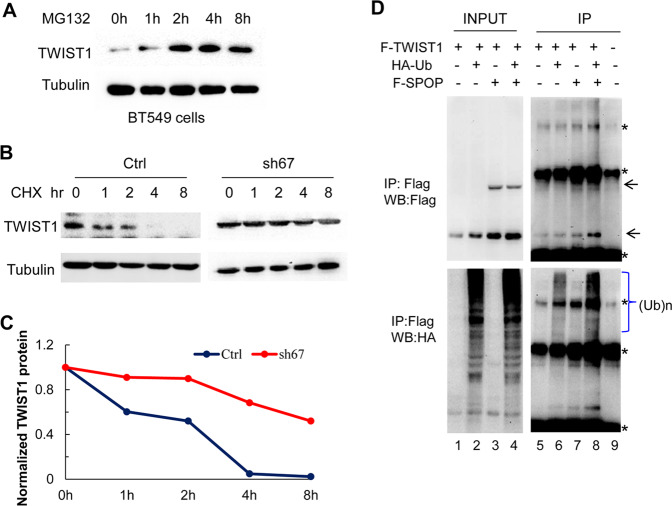
SPOP ubiquitinates TWIST1 and destabilizes it. **A** MG132 stabilizes TWIST1. **B** Knock-down of SPOP prolongs the half-life of TWIST1 protein. Cell lines transfected with shCtrl or shSPOP (sh67) vectors were treated with 20 µg/ml CHX and whole protein lysates were collected at the indicated times for western blotting. **C** TWIST1 protein level was quantified and plotted relative to the 0-time point. **D** SPOP ubiquitinates TWIST1. HeLa cells with transient overexpression of indicated either HA-Ubiquitin (UA-Ub), FLAG-SPOP (F-SPOP), FLAG-TWIST1 (F-TWIST1) or combination were harvested and subject to M2 beads immune-precipitation (IP: FLAG) and western blotting with indicated antibodies.

The inverse correlation between SPOP and TWIST1 in breast carcinoma tissues and their functional interplay prompted us to address the regulatory mechanisms. To test if the proteasome system is critical in the regulation of TWIST1 stability, we treated BT549 cells with a proteasome inhibitor, MG132, for various duration followed by western blotting analysis. Blockage of proteasome function led to accumulation of TWIST1 (Fig. 4A). Cells transfected shCtrl or shSPOP (sh67) vectors were treated with 20 µg/ml CHX for indicated times and assayed for dynamic changes in TWIST1. Prevention of de novo protein synthesis by CHX revealed a much faster degradation of TWIST1 in control (shCtrl) cells than in shSPOP (sh67)-treated cells (Fig. 4B), indicating that silencing SPOP stabilized TWIST1 and increased its half-life from ~2 h to nearly 8 h (Fig. 4B, C). Consistent with these findings, in vivo analysis demonstrates that SPOP promoted TWIST1 poly-ubiquitination (Fig. 4D).

**Fig. 5:**
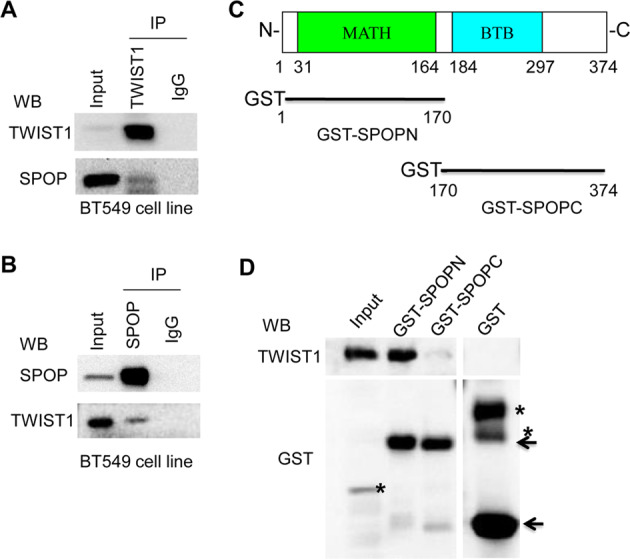
SPOP interacts with TWIST1. Endogenous SPOP associated with endogenous TWIST1. TWIST1 precipitated (**A**) and co-precipitated (**B**) with SPOP. **C** Schematic illustration of GST-tagged C-terminal deletion mutant (GST-SPOPN) and N-terminal deletion mutant (GST-SPOPC) of SPOP. **D** N-terminal region of SPOP interacts with TWIST1 directly.

To understand whether SPOP-mediated TWIST1 degradation is through direct SPOP and TWIST1 interaction, we carried out immuno-precipitation (IP) and GST pull-down assays. Intracellular interactions between SPOP and TWIST1 were shown by immuno-precipitation (IP) with a TWIST1 antibody (Fig. 5A) or an SPOP antibody (Fig. 5B). To ensure direct physical interactions between SPOP and TWIST1, SPOP subclones containing either the MATH domain or BTB domain (Fig. 5C) were used for GST pull-down assays. The results demonstrate that only the N-terminal region (with MATH domain) of SPOP interacted with TWIST1 in vitro (Fig. 5C, D). These findings substantiated that SPOP destabilizes TWIST1 via direct interaction.

### SPOP enhances K63- and K48-linked ubiquitination of TWIST1

**Fig. 6:**
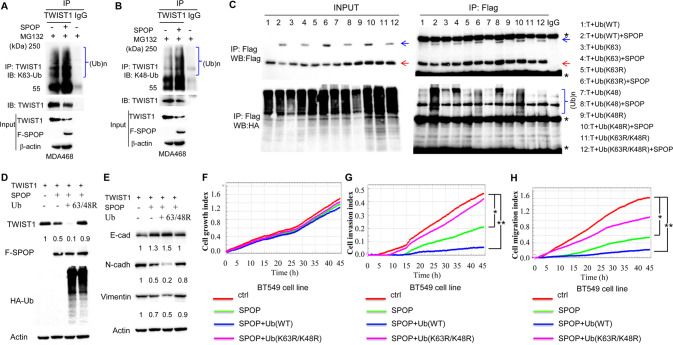
SPOP promotes both K63-linked and K48-linked polyubiquitination of TWIST1. **A** SPOP promotes K63-linked ubiquitination (K63-Ub) of TWIST1. **B** SPOP promotes K48-linked ubiquitination (K48-Ub) of TWIST1. MDA-MB-468 cells (MDA468) were serum-starved, treated with MG132 for 2 h and lysed cells for IP with TWIST1 antibody, followed by IB analysis with indicated antibodies. **C** Both sites of K48 and K63 in ubiquitin are required for SPOP to promote ubiquitination of TWIST1. In vivo ubiquitination assay in TWIST1-inducible overexpressed cell transfected with Flag-tagged-SPOP along with MG132 treatment. T, TWIST1. Lane 1: TWIST1 + wild type ubiquitin Ub(WT), lane 2: TWIST1 + Ub(WT) + SPOP, lane 3: TWIST1 + Ub(K63), lane 4: TWIST1 + Ub(K63) + SPOP, lane 5: TWIST1 + Ub(K63R), lane 6:TWIST1 + Ub(K63R) + SPOP, lane 7:TWIST1 + Ub(K48), lane 8:TWIST1 + Ub(K48) + SPOP, lane 9:TWIST1 + Ub(K48R), lane 10: TWIST1 + Ub(K48R) + SPOP, lane 11:TWIST1 + Ub(K63R/K48R), lane 12:TWIST1 + Ub(K63R/K48R) + SPOP, lane IgG: IP with IgG. Red arrows, TWIST1, blue arrows, SPOP, and stars “*”, non-specific. **D** Double mutations of both K63-linked and K48-linked ubiquitin Ub-K63R/K48R (63/48R) attenuated SPOP promoted TWIST1 degradation. TWIST1 inducible overexpressed cells were transfected with or without Flag-tagged-SPOP along with HA-Ub or double mutations of both K63-linked and K48-linked ubiquitination Ub-K63R/K48R (63/48R), and protein lysates were collected for western blotting analysis after doxycycline (Dox) treatments for one day. **E** Double mutations of both K63-linked and K48-linked ubiquitination Ub-K63R/K48R (63/48R) on TWIST1-attenuated SPOP promoted EMT marker regulation by stabilizing TWIST1. The same protein lysates from Fig. **D** were used for western blotting analysis. Double mutations of K63-linked and K48-linked ubiquitination Ub-K63R/K48R (63/48R) on TWIST1 attenuated SPOP promoted cell growth (**F**), invasion (**G**) and migration (**H**). BT549 cells were transfected with Flag-tagged TWIST1 with or without Flag-tagged-SPOP along with HA-Ub or double mutations of both K63-linked and K48-linked ubiquitination Ub-K63R/K48R (63/48R), and real-time cell growth index, migration and invasion assays were performed in a real time cell analyzer. All experiments were repeated at least three times. “*” refers to *p*-value ≤ 0.05 and “**” for *p*-value ≤ 0.01.

It is known that in the presence of MG132, RNF8 promotes K63- and K48-linked ubiquitination of TWIST1 [42, 43], whereas EGF only induces K63-linked ubiquitination of intracellular TWIST1 [43]. To determine the mode of lysine ubiquitination by SPOP, we overexpressed Flag-tagged SPOP or control in MDA-MB-468 (MDA468) cells in the presence of MG132, followed by immunoprecipitation (IP) with a TWIST1 antibody and western blotting by an antibody against K63 -specific polyubiquitin. In the control experiments, EGF treatments induced K63-linked ubiquitination of TWIST1 in the BT549 breast cancer cell line (Supplementary Fig. S5A). SPOP overexpression dramatically enhanced K63-linked ubiquitination of TWIST1 (Fig. 6A, middle lane in top panel, Supplementary Fig. S5B). Similar in vivo ubiquitination assays performed with an antibody against K48 linkage-specific polyubiquitin disclosed accumulation of K48-linked ubiquitination of TWIST1 (Fig. 6B, middle lane of top panel, Supplementary Fig. S5C).

To clarify the detailed ubiquitination mechanisms, we overexpressed HA-tagged wild-type or different mutant forms of ubiquitin (UB-WT, UB-K63, UB-K63R, UB-K48, UB-K48R, and double mutation for UB-K63R/K48R), along with Flag-tagged SPOP or controls in a cell line inducible expressing TWIST1 [33]. The results demonstrate that K63-linked polyubiquitination is the major type of ubiquitin chain topology for the modification of TWIST1 by SPOP (Fig. 6C, lanes 5 & 6 vs. lanes 3 & 4 in the right of bottom panel), followed by K48-linked polyubiquitination (Fig. 6C, lanes 9 & 10 vs. lanes 7 & 8 in the right of bottom panel). In contrast to wild type Ub (WT) (Fig. 6C, lane 2 vs. 1 in the right of bottom panel), double mutations in Ub (UB-K63R/K48R) completely erased TWIST1 ubiquitination by SPOP (Fig. 6C, lanes 11 & 12 vs. lane IgG in the right of bottom panel). These results demonstrate that SPOP promotes K63- and K48-linked ubiquitination of TWIST1.

### Ub-K63R/K48R double mutant cells are resistant to SPOP mediated tumor suppression

Given that Ub-K63R/K48R double mutations abolish TWIST1 ubiquitination by SPOP, we investigated whether cells with Ub-K63R/K48R double mutations might eliminate SPOP induced TWIST1 degradation and subsequent biological changes. As expected, overexpression of Ub(WT) together with SPOP reduced TWIST1 protein level 5-fold compared with overexpression of SPOP only (Fig. 6D, lane 2 vs. lane 3). Upon overexpression of the Ub-K63R/K48R double mutation, TWIST1 protein levels dramatically increased 9-fold compared to cells expressing Ub(WT), despite high SPOP levels (Fig. 6D, lane 4 vs. lane 3). Stabilized TWIST1 in Ub-K63R/K48R double mutation cells induced a reduction in E-cadherin (Fig. 6E, lane 3 vs. lane 4), and an increase in N-cadherin (Fig. 6E, lane 3 vs. lane 4) and Vimentin (Fig. 6E, lane 3 vs. lane 4). These results demonstrate that Ub-K63R/K48R double mutations attenuate SPOP dependent degradation of TWIST1 with changes in corresponding EMT markers in TNBC cells.

Then, we examined the impact of Ub-K63R/K48R double mutations on invasion and migration capability in TNBC cells. When Ub-K63R/K48R and SPOP were overexpressed in BT549, the cell growth index was not significantly changed (Fig. 6F). However, Ub-K63R/K48R/SPOP expressing cells displayed much more enhanced capabilities in cell invasion and migration than Ub(WT)/SPOP expressing cells (Fig. 6G, H, pink lines vs. blue lines), even higher than in cells overexpressing SPOP only (Fig. 6G, H, pink lines vs. green lines). These observations were further endorsed in the HEK293-TWIST1 inducible cell line (Supplementary Fig. S6) where the same invasion and migration actions exist due to the Ub-K63R/K48R/SPOP expression patterns.

### Lysine residue K73 in TWIST1 is the site for ubiquitination

**Fig. 7:**
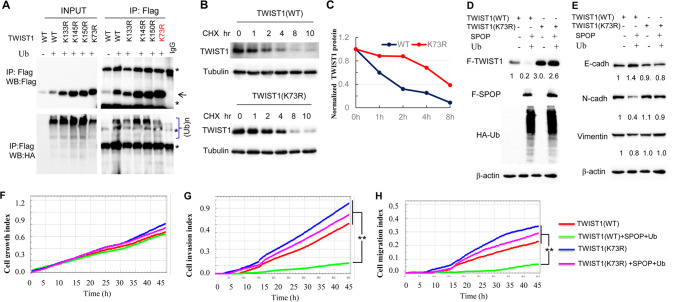
K73 of TWIST1 is the major ubiquitinated site and K73R TWIST1 is resistant to SPOP degradation. **A** In vivo ubiquitination assays in HeLa cells transfected with Flag-tagged wild-typeTWIST1(WT), its mutant forms K133R, K145R, K150R, and K73R, respectively, along with HA-tagged ubiquitin (HA-Ub) constructs. This was followed by lysis of cells for IP with TWIST1 antibody, followed by IB analysis with indicated antibodies. **B**, **C** The protein half-life of K73R mutated TWIST1(K73R) was prolonged. Flag-tagged both wild-type and K73R mutated TWIST1 inducible overexpressed cells were treated with 20 µg/ml CHX after doxycycline (Dox) treatments for one day, and protein lysates were collected at the indicated times for western blotting. **D** K73R of TWIST1 is resistant to SPOP degradation. Flag-tagged wild-type or K73R mutated TWIST1 inducible overexpressed cells were transfected with or without Flag-tagged-SPOP along with HA-Ub, and protein lysates were collected for western blotting analysis. **E** K73R of TWIST1-attenuated SPOP promoted EMT marker regulation. The same protein lysates from Fig. **D** were used for western blotting analysis. K73R of TWIST1 attenuated SPOP promoted cell growth (**F**), invasion (**G**) and migration (**H**). Flag-tagged wild-type or K73R mutated TWIST1 inducible overexpressed cells were transfected with or without Flag-tagged-SPOP along with HA-Ub, and real-time cell growth index, migration and invasion assays were performed in a real time cell analyzer. All experiments were repeated at least three times. *P*-value ≤ 0.01 was considered as a significant difference (**).

To understand molecular details of TWIST1 ubiquitination, we performed LC-MS/MS analysis of purified Flag-tagged TWIST1 and identified four lysine residues that were modified by ubiquitination, including K73, K133, K145, and K150 (Table 1, Supplementary Fig. S7). To distinguish the contribution of these sites, we generated TWIST1 clones with K133R, K145R, K150R, or K73R mutations, respectively. Intracellular ubiquitination assays were conducted in HeLa cells by transfecting a Flag-tagged wild-type TWIST1 (WT) or various mutant TWIST1 (K133R, K145R, K150R, or K73R), along with HA-tagged ubiquitin (HA-Ub) constructs. Following IP with anti-Flag and western blotting analysis with anti-Flag or anti-HA antibodies, we found that K73 of TWIST1 was the major ubiquitinated site due to the significantly reduced amount of TWIST1 ubiquitination detected (Fig. 7A, lanes 6 with red color in the right bottom of panels). Although K133 TWIST1 also had reduced amount of TWIST1 ubiquitination (Fig. 7A, lanes 3 in the right bottom of panel), the amount of TWIST1 by IP in lanes 3 in the right upper of panel was much less than other mutant TWIST1 (Fig. 7A, lanes 3 in the right upper of panel), suggesting a “loading” issue. Therefore, K133 in TWIST1 may not be a bona fide ubiquitination site. Taken together, we conclude that K73 of TWIST1 is the major site for ubiquitination, with K133 as an alternative option.

**Table 1:**
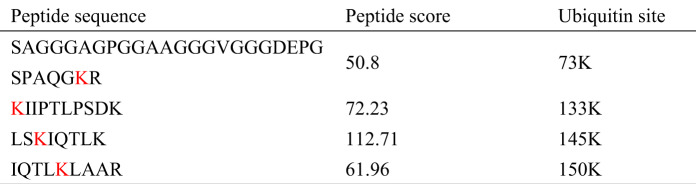
List and site details of ubiquitinated peptides in TWIST1.

Note: K in red indicates ubiquitin sites.

### K73 in TWIST1 is critical for TWIST1 stabilization and cancer malignancy

Then, we endeavored to dissect the action of K73R mutation in TWIST1 in protein stabilization and related pathological action. We engineered a HEK293-TWIST1(K73R) inducible cell line, and analyzed degradation dynamics in the presence of CHX. The results clearly demonstrated that cells with the K73R mutation had more significant accumulation of TWIST1 than cells with wild-type TWIST1(WT), suggesting a prolonged half-life for the TWIST1 K73R mutant protein (Fig. 7B, C). This was supported by the observation that the TWIST1(K73R) mutant is resistant to SPOP induced degradation regardless of the polyubiquitination chain status (Fig. 7D, compare line 3 vs. lane 1; line 3 vs. lane 4). As expected, overexpression of TWIST1(K73R) overrode SPOP-mediated changes in EMT markers, including E-cadherin, N-cadherin, and Vimentin (Fig. 7E, lane 2 vs. lane 4; lane 3 vs. lane 4).

Finally, we examined the impact of TWIST1(K73R) on invasion and migration of the cancer cells. Upon overexpression of TWIST1(K73R), together with SPOP, the cell growth index was barely affected (Fig. 7F). However, the capabilities of cell invasion and migration were significantly enhanced, compared with overexpression of TWIST1(WT) and SPOP (Fig. 7G, H, pink lines vs. green lines). Strikingly, the capabilities of cell invasion and migration were highest when cells were overexpressing TWIST1(K73R) alone (Fig. 7G, H, blue lines vs. pink lines, red or green lines). We conclude that K73 of TWIST1 is critical for TWIST1 stabilization, thus promoting cancer cell migration and invasion.

## Discussion

SPOP has been demonstrated a tumor suppresser. Cancer genome analysis has identified recurrent missense mutations in the *SPOP* gene in 11~13% of primary prostate cancer (PC) [20–22] and in 6~8% of metastatic and castration-resistant PC [23]. As an important member of the E3 ubiquitin- ligase complex [17, 18], most studies on SPOP mainly focused on PC but not on breast cancer [18, 20, 22, 23, 25]. While SPOP functions in breast cancer tumorigenesis and progression are not fully determined [31], Li et al first demonstrate that SPOP interacts directly with SRC-3 in a phosphorylation-dependent manner, thus targeting SRC-3 in breast cancers. Breast cancer metastasis suppressor 1 (BRMS1) [44], progesterone receptor (PR) [45] and c-Myc [46] have been indicated as direct targets of SPOP in breast cancer for ubiquitin-dependent proteasomal degradation. Little is known regarding the *SPOP* gene mutations in Chinese breast cancer patients so far [30], implying alternative mechanisms in breast cancer progression, particularly for TNBC and advanced cancer stages [45].

Our and other previous studies have revealed important roles and mechanisms of TWIST1 in breast cancer metastasis [8, 9, 33]. Based on the TCGA analysis that there is no correlation between TWIST1 expression and amplified CNV, we believe that there is an unknown regulatory mechanism for TWIST1 at the post-transcriptional level. In the current study, we found that SPOP destabilizes TWIST1 through the ubiquitin proteasomal pathway, thereby inhibiting EMT and breast cancer metastasis in vitro and in vivo. Moreover, lower SPOP and higher TWIST1 are correlated with poor prognosis in breast cancer patients. With its tumor suppressive function, SPOP may prevent breast cancer progression into the advanced stage, particularly in TNBC. TNBC or advanced breast cancers are considered generally more aggressive than other breast carcinomas with high risks of recurrences, lower 5-year survival after diagnosis, and limited therapeutic approaches. Therefore, large cohorts of SPOP/TWIST1 studies in TNBC patients provide additional relevant predictors of survival, mortality and tumor recurrence. In the aggregate, these results suggest that the outcomes of SPOP on breast cancer malignancy are mediated, at least in part, by TWIST1.

Our studies revealed several unexpected findings with significant implications. We have discovered a correlation between SPOP and TWIST1 in TNBC and defined a vital role of SPOP-mediated K63-linked and K48-linked ubiquitination of TWIST1 in TNBC where TWIST1 triggers EMT, cell migration and invasion. Our observation of K63-linked and K48-linked ubiquitination reflects important post-translational regulatory mechanisms in control of TWIST1. Moreover, lysine residue 73 (K73) in human TWIST1 is identified as the critical site for ubiquitination that modulates protein homeostasis, cancer cell growth, migration and invasion of TNBC.

**Fig. 8:**
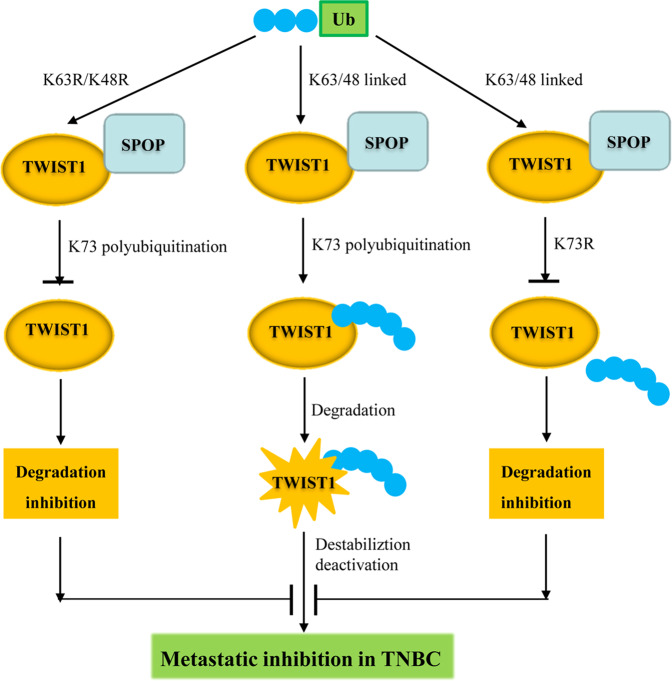
A working mechanistic model underlying SPOP mediated TWIST1 degradation and metastasis inhibition. SPOP mediated TWIST1 degradation and metastasis inhibition can be modulated by K63-linked and K48-linked TWIST1 ubiquitination at site K73; either mutated K63R/K48R of Ub or mutated K73R of TWIST1 disrupted SPOP-mediated TWIST1 ubiquitination and its further depredation, thereby blocking metastasis inhibition in TNBC.

A working model underlying the mechanistic action of SPOP on TWIST1 and cancer metastasis is depicted in Fig. 8. In this model, K63-linked and K48-linked polyubiquitination at K73 in TWIST1 trigger its degradation by SPOP, thereby preventing EMT and subsequent cell migration and invasion. K63R/K48R double mutations in Ub or K73R mutation in TWIST1 prevent TWIST1 degradation, promoting EMT, cell migration and invasion, the phenotypes characteristic of TNBC (Fig. 8).

Previous studies have reported that several kinases can phosphorylate TWIST1 [47–50]. TWIST1 phosphorylation reduces TWIST1 protein levels by recruiting FBXL14/Ppa and/or β-TRCP E3 ligases, which target TWIST1 for K48-linked ubiquitination and subsequent degradation [48, 50]. The clinically available AKT1 inhibitor, MK-2206, can stabilize TWIST1 and enhance EMT and metastasis in breast cancer cells. To our knowledge, K48-linked ubiquitination is always responsible for target degradation but K63-linked ubiquitination might be a signal for target activation/stablility. In addition, Lee et al have reported that RNF8-promoted K63-linked ubiquitination triggers trans-localization of cellular TWIST1 to nucleus to promote EMT and cancer stem cell (CSC) phenotypes, suggesting that K63-linked ubiquitination might be a signal for activation of TWIST1 [43]. K63-linked ubiquitination may also prevent proteasome-mediated degradation on TWIST1 [43]. Detailed mechanisms underlying the controversial actions between K63- and K48-linked ubiquitination of TWIST1 need further investigation.

Besides ubiquitination, other post-translational modifications, e.g., methylation and acetylation, occur at lysine (K) residues. Although methylation at K residues of non-histone proteins has emerged as a prevalent post-translational modification and as a vital regulator in signal transduction [51], there is no report of lysine methylation of TWIST1. Shi et al have reported that TWIST1 is acetylated at both K73 and K76 [52]. TWIST1 acetylation recruits BRD4/P-TEFb/RNA-Pol II to activate *Wnt5A* expression and subsequent Wnt5a-mediated EMT processes. Consistent with our observation, TWIST1 modification (acetylation) does not influence cellular-nuclear transportation of TWIST1. Their study together with ours indicates that different types of lysine modifications mediate TWIST1 actions via distinct mechanisms. The detailed mechanism underlying the crosstalk between ubiquitination and acetylation of TWIST1 at K73 requires further investigation.

Taken together, we have linked SPOP to TWIST1 destabilization for its tumor suppressive functions such as inhibiting EMT and metastasis of breast cancer cells. The SPOP-TWIST1 pathway disclosed in this study may provide potentially novel therapeutics and prognostic markers for advanced breast cancer patients.

## Supplementary information

Supplementary figures

Raw data for original western blots
