# Supplementary material for: The speckle-type POZ protein (SPOP) inhibits breast cancer malignancy by destabilizing TWIST1
Source: Cell Death Discov. 2022 Sep 17;8:389. doi: 10.1038/s41420-022-01182-3 (PMC9482615; doi:10.1038/s41420-022-01182-3)
Supplement: Supplementary file 1 — Supplementary figures [file 41420_2022_1182_MOESM1_ESM.ppt]

## Slide 1
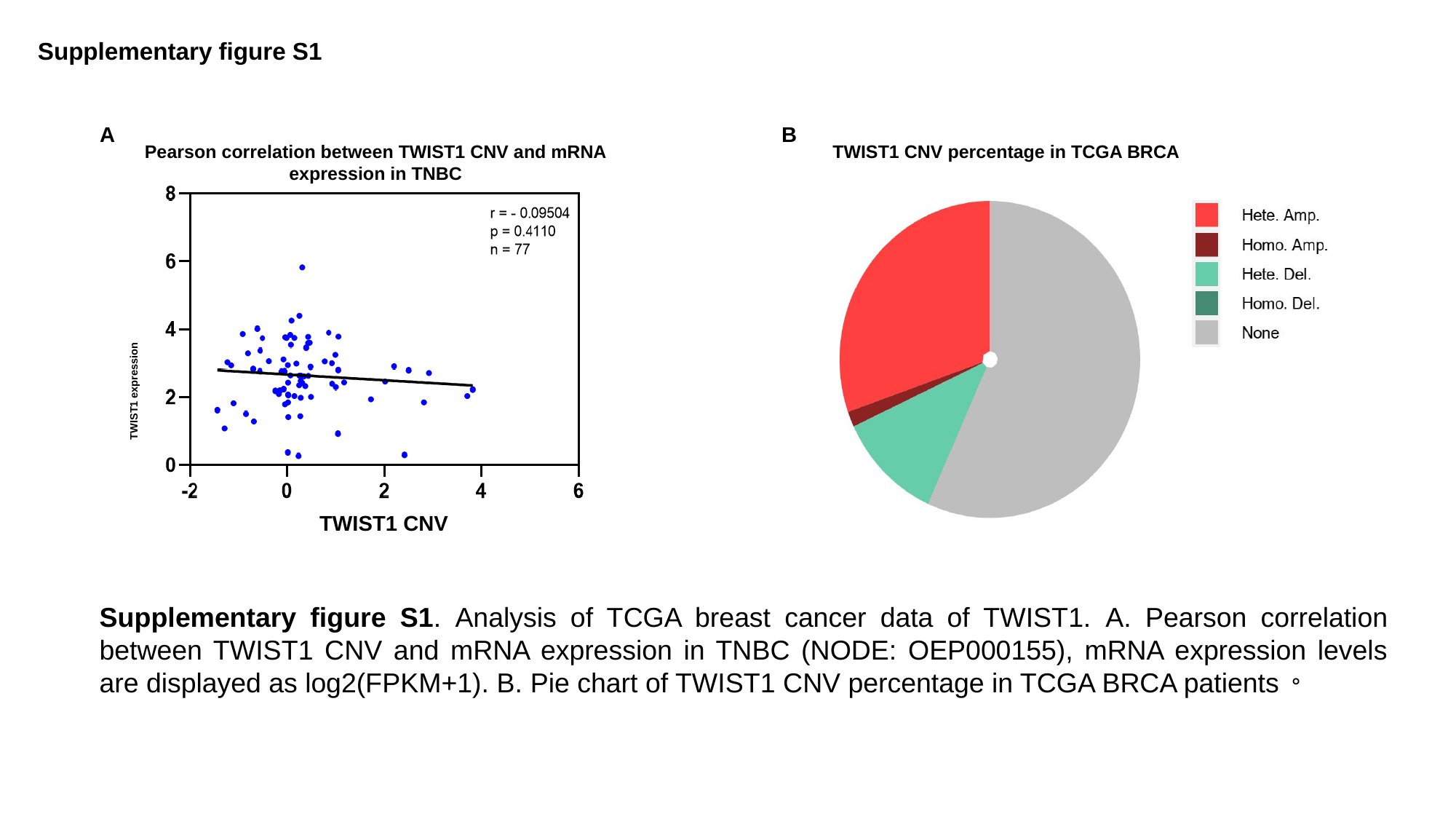

Supplementary figure S1
A
B
Pearson correlation between TWIST1 CNV and mRNA expression in TNBC
TWIST1 expression
TWIST1 CNV
TWIST1 CNV percentage in TCGA BRCA
Supplementary figure S1. Analysis of TCGA breast cancer data of TWIST1. A. Pearson correlation between TWIST1 CNV and mRNA expression in TNBC (NODE: OEP000155), mRNA expression levels are displayed as log2(FPKM+1). B. Pie chart of TWIST1 CNV percentage in TCGA BRCA patients。

## Slide 2
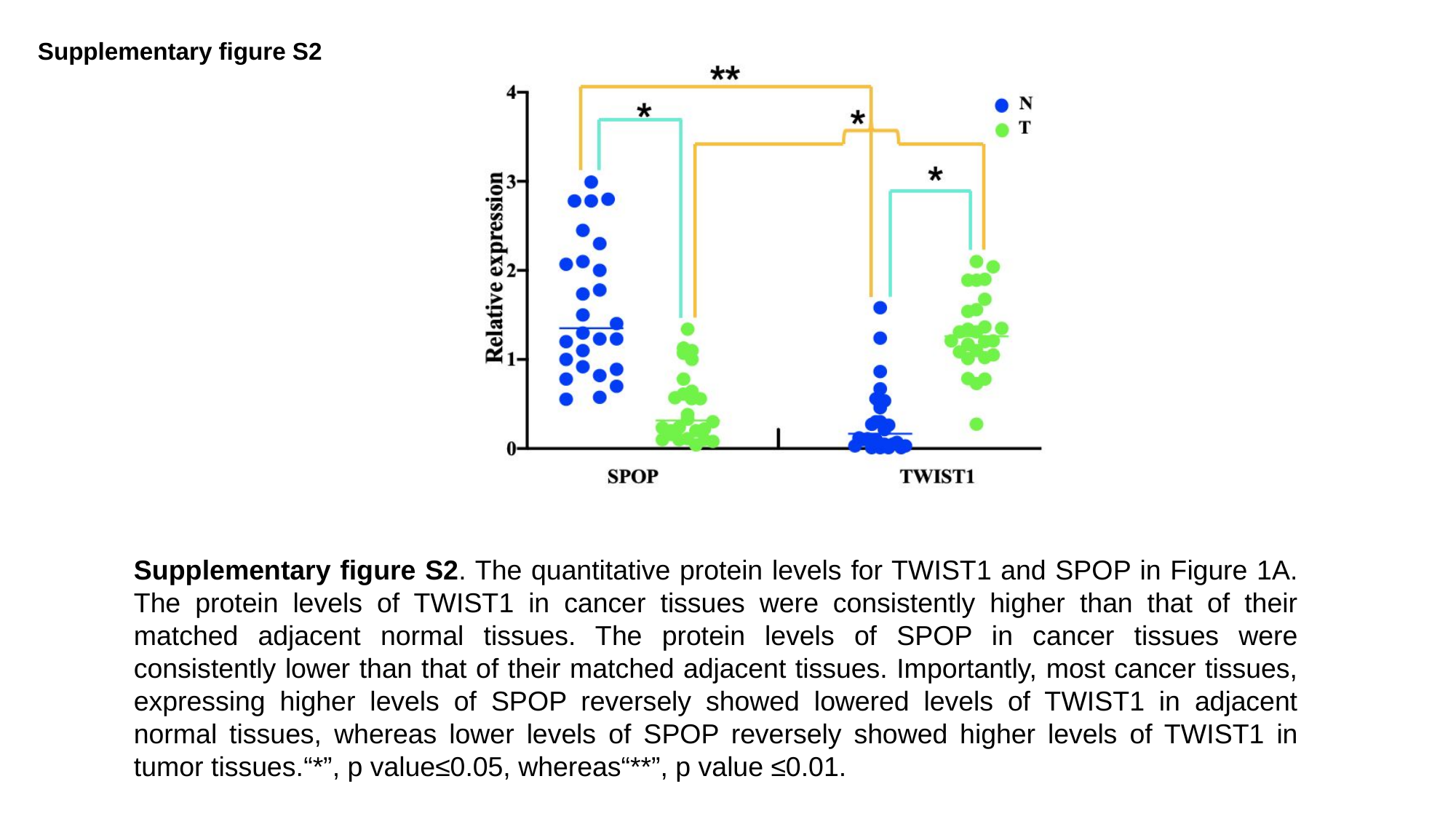

Supplementary figure S2
Supplementary figure S2. The quantitative protein levels for TWIST1 and SPOP in Figure 1A. The protein levels of TWIST1 in cancer tissues were consistently higher than that of their matched adjacent normal tissues. The protein levels of SPOP in cancer tissues were consistently lower than that of their matched adjacent tissues. Importantly, most cancer tissues, expressing higher levels of SPOP reversely showed lowered levels of TWIST1 in adjacent normal tissues, whereas lower levels of SPOP reversely showed higher levels of TWIST1 in tumor tissues.“*”, p value≤0.05, whereas“**”, p value ≤0.01.

## Slide 3
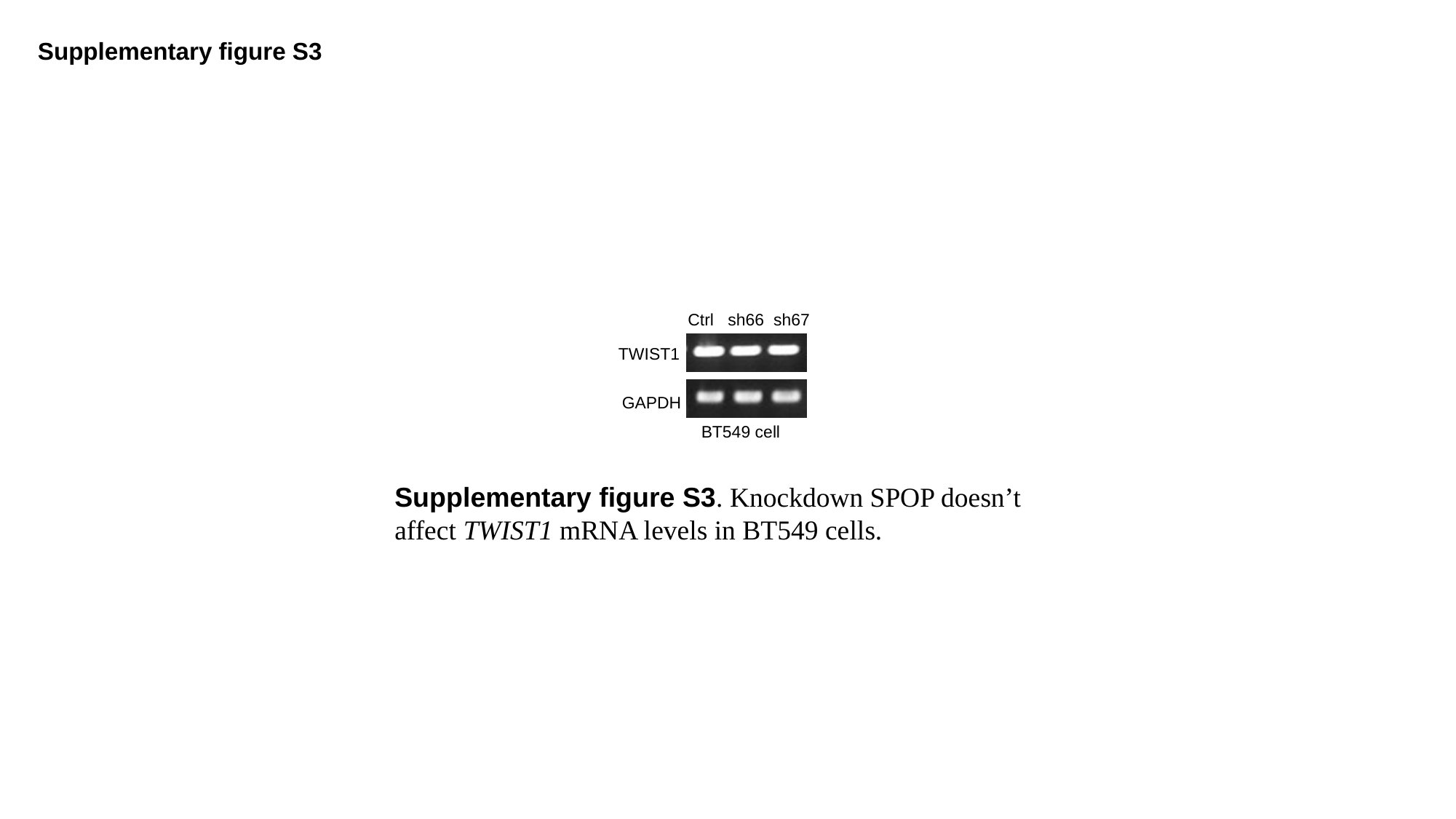

Supplementary figure S3
Ctrl sh66 sh67
TWIST1
GAPDH
BT549 cell
Supplementary figure S3. Knockdown SPOP doesn’t affect TWIST1 mRNA levels in BT549 cells.

## Slide 4
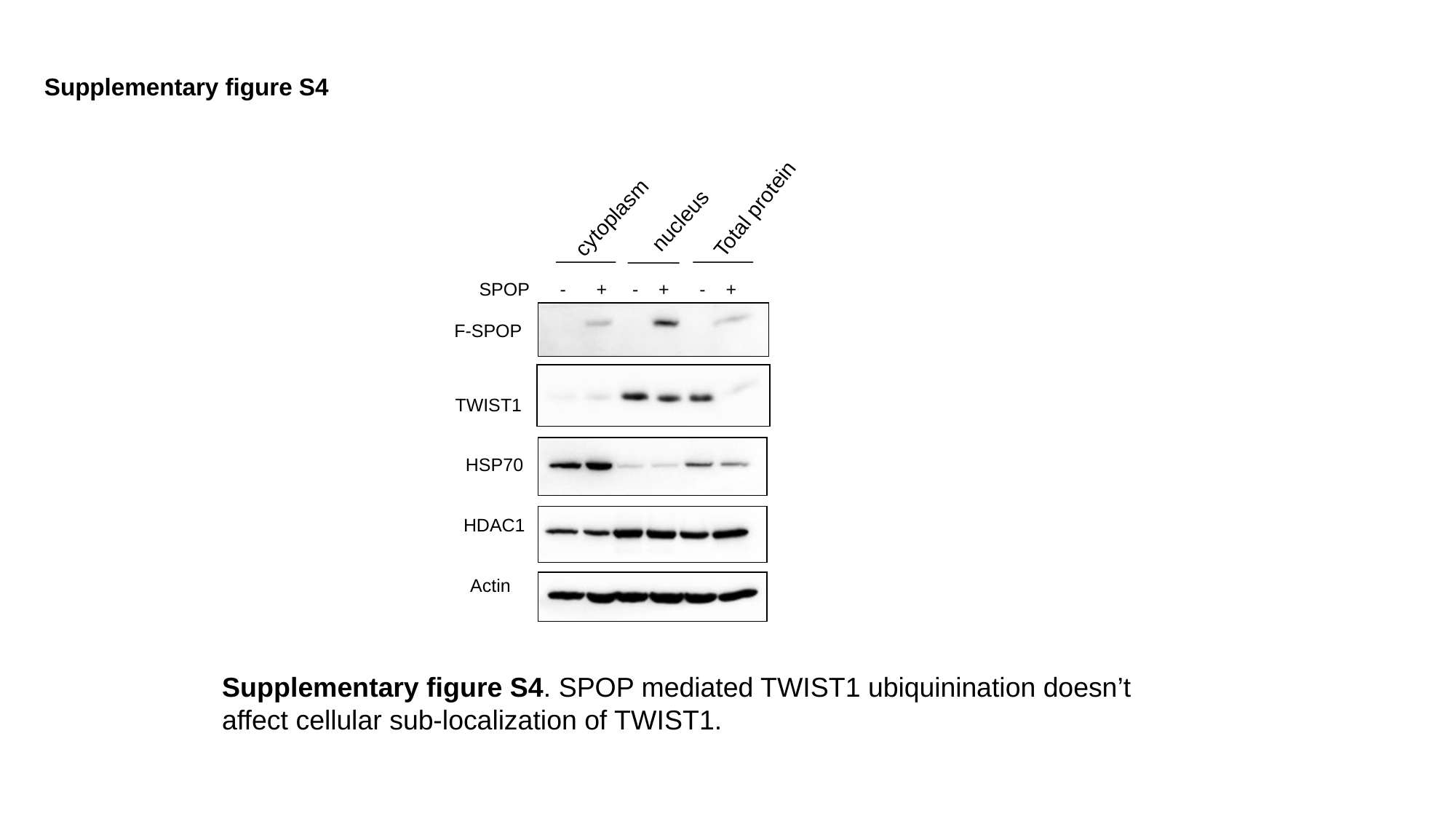

Supplementary figure S4
cytoplasm
nucleus
SPOP - + - + - +
F-SPOP
TWIST1
HSP70
HDAC1
Actin
Total protein
Supplementary figure S4. SPOP mediated TWIST1 ubiquinination doesn’t affect cellular sub-localization of TWIST1.

## Slide 5
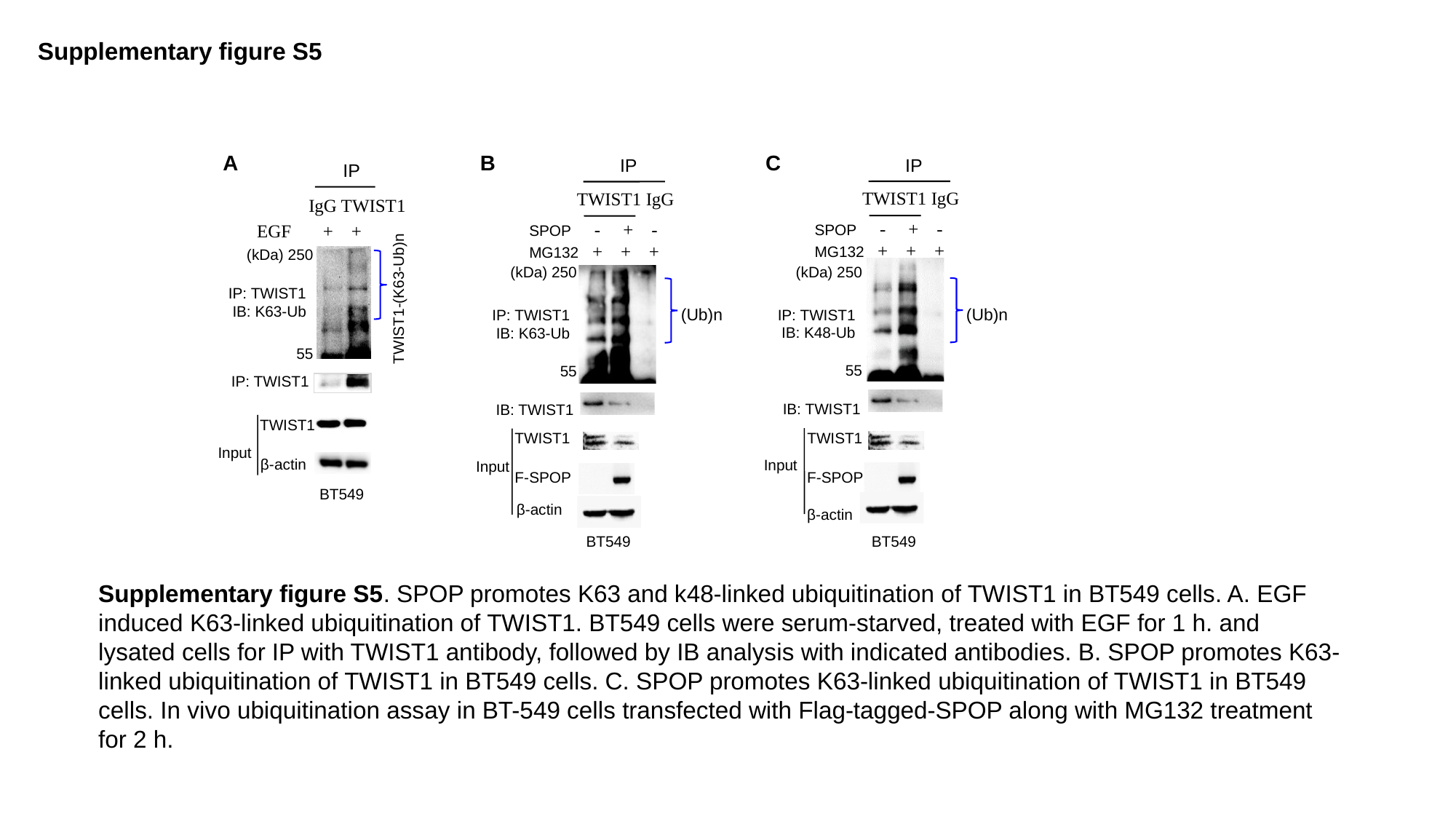

Supplementary figure S5
A
IP
IgG TWIST1
EGF + +
(kDa) 250
55
IP: TWIST1
IB: K63-Ub
TWIST1-(K63-Ub)n
IP: TWIST1
Input
β-actin
BT549
C
IP
TWIST1 IgG
SPOP - + -
MG132 + + +
(kDa) 250
55
(Ub)n
IP: TWIST1
IB: K48-Ub
IB: TWIST1
TWIST1
Input
F-SPOP
β-actin
BT549
B
IP
TWIST1 IgG
SPOP - + -
MG132 + + +
(kDa) 250
55
(Ub)n
IP: TWIST1
IB: K63-Ub
IB: TWIST1
TWIST1
Input
F-SPOP
β-actin
BT549
TWIST1
Supplementary figure S5. SPOP promotes K63 and k48-linked ubiquitination of TWIST1 in BT549 cells. A. EGF induced K63-linked ubiquitination of TWIST1. BT549 cells were serum-starved, treated with EGF for 1 h. and lysated cells for IP with TWIST1 antibody, followed by IB analysis with indicated antibodies. B. SPOP promotes K63-linked ubiquitination of TWIST1 in BT549 cells. C. SPOP promotes K63-linked ubiquitination of TWIST1 in BT549 cells. In vivo ubiquitination assay in BT-549 cells transfected with Flag-tagged-SPOP along with MG132 treatment for 2 h.

## Slide 6
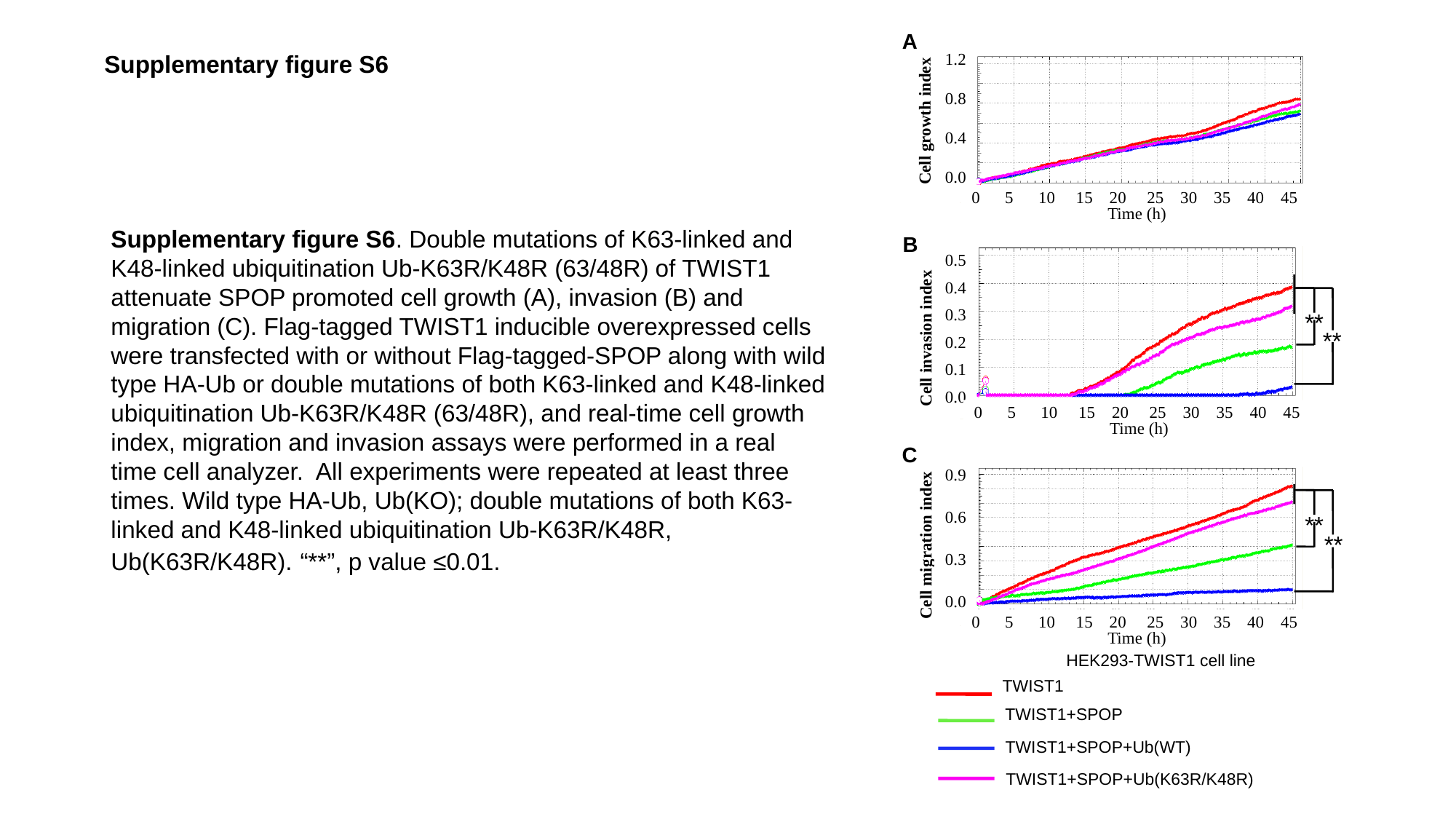

A
1.2
0.8
0.4
0.0
Cell growth index
0 5 10 15 20 25 30 35 40 45
 Time (h)
B
0.5
0.4
0.3
0.2
0.1
0.0
 Cell invasion index
0 5 10 15 20 25 30 35 40 45
 Time (h)
C
 Cell migration index
0.9
0.6
0.3
0.0
0 5 10 15 20 25 30 35 40 45
 Time (h)
HEK293-TWIST1 cell line
TWIST1
TWIST1+SPOP
TWIST1+SPOP+Ub(WT)
TWIST1+SPOP+Ub(K63R/K48R)
Supplementary figure S6
Supplementary figure S6. Double mutations of K63-linked and K48-linked ubiquitination Ub-K63R/K48R (63/48R) of TWIST1 attenuate SPOP promoted cell growth (A), invasion (B) and migration (C). Flag-tagged TWIST1 inducible overexpressed cells were transfected with or without Flag-tagged-SPOP along with wild type HA-Ub or double mutations of both K63-linked and K48-linked ubiquitination Ub-K63R/K48R (63/48R), and real-time cell growth index, migration and invasion assays were performed in a real time cell analyzer. All experiments were repeated at least three times. Wild type HA-Ub, Ub(KO); double mutations of both K63-linked and K48-linked ubiquitination Ub-K63R/K48R, Ub(K63R/K48R). “**”, p value ≤0.01.
**
**
**
**

## Slide 7
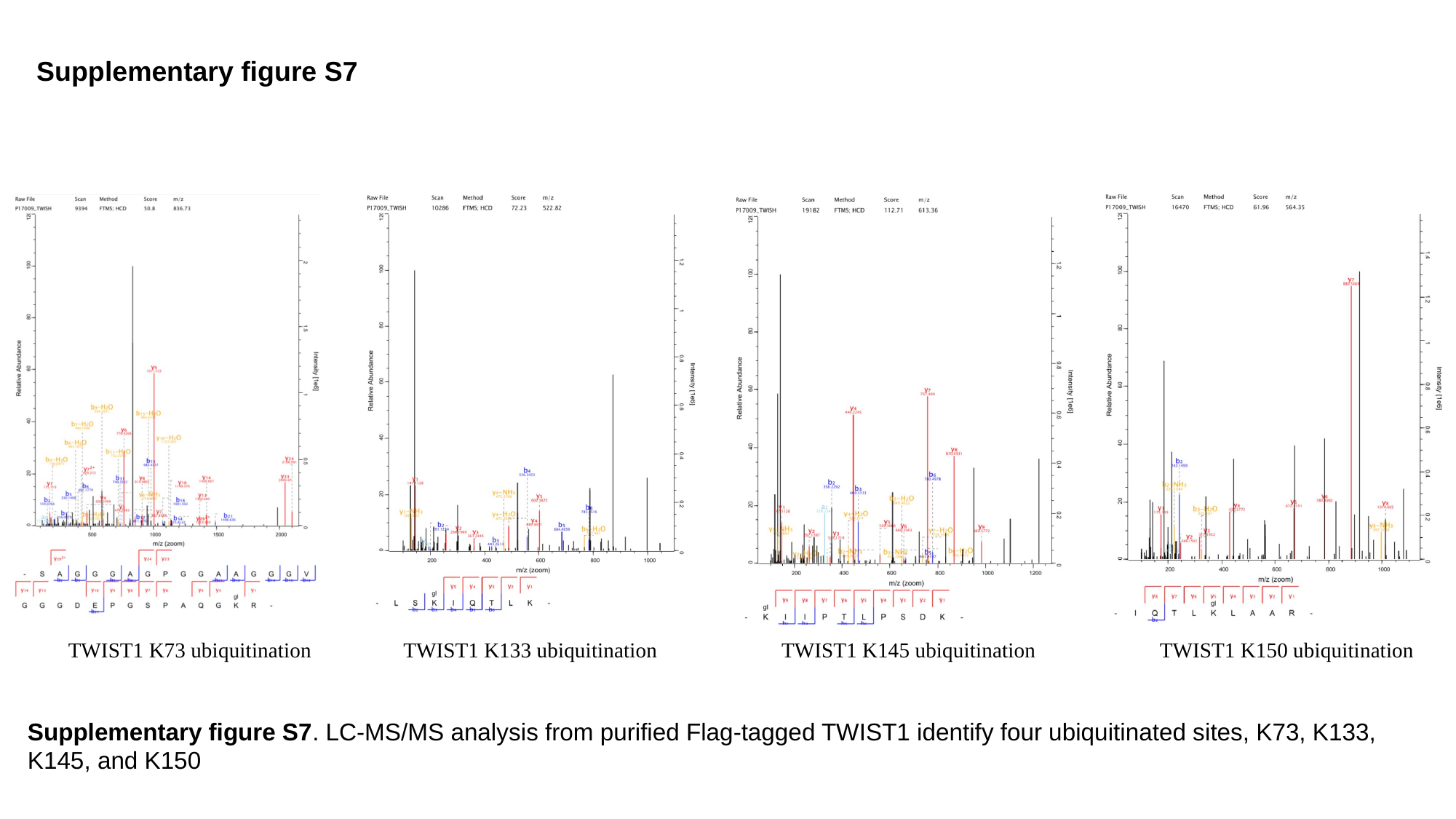

Supplementary figure S7
TWIST1 K73 ubiquitination
TWIST1 K133 ubiquitination
TWIST1 K145 ubiquitination
TWIST1 K150 ubiquitination
Supplementary figure S7. LC-MS/MS analysis from purified Flag-tagged TWIST1 identify four ubiquitinated sites, K73, K133, K145, and K150
