# Supplementary material for: The speckle-type POZ protein (SPOP) inhibits breast cancer malignancy by destabilizing TWIST1
Source: Cell Death Discov. 2022 Sep 17;8:389. doi: 10.1038/s41420-022-01182-3 (PMC9482615; doi:10.1038/s41420-022-01182-3)
Supplement: Supplementary file 2 — Raw data for original western blots [file 41420_2022_1182_MOESM2_ESM.ppt]

## Slide 1
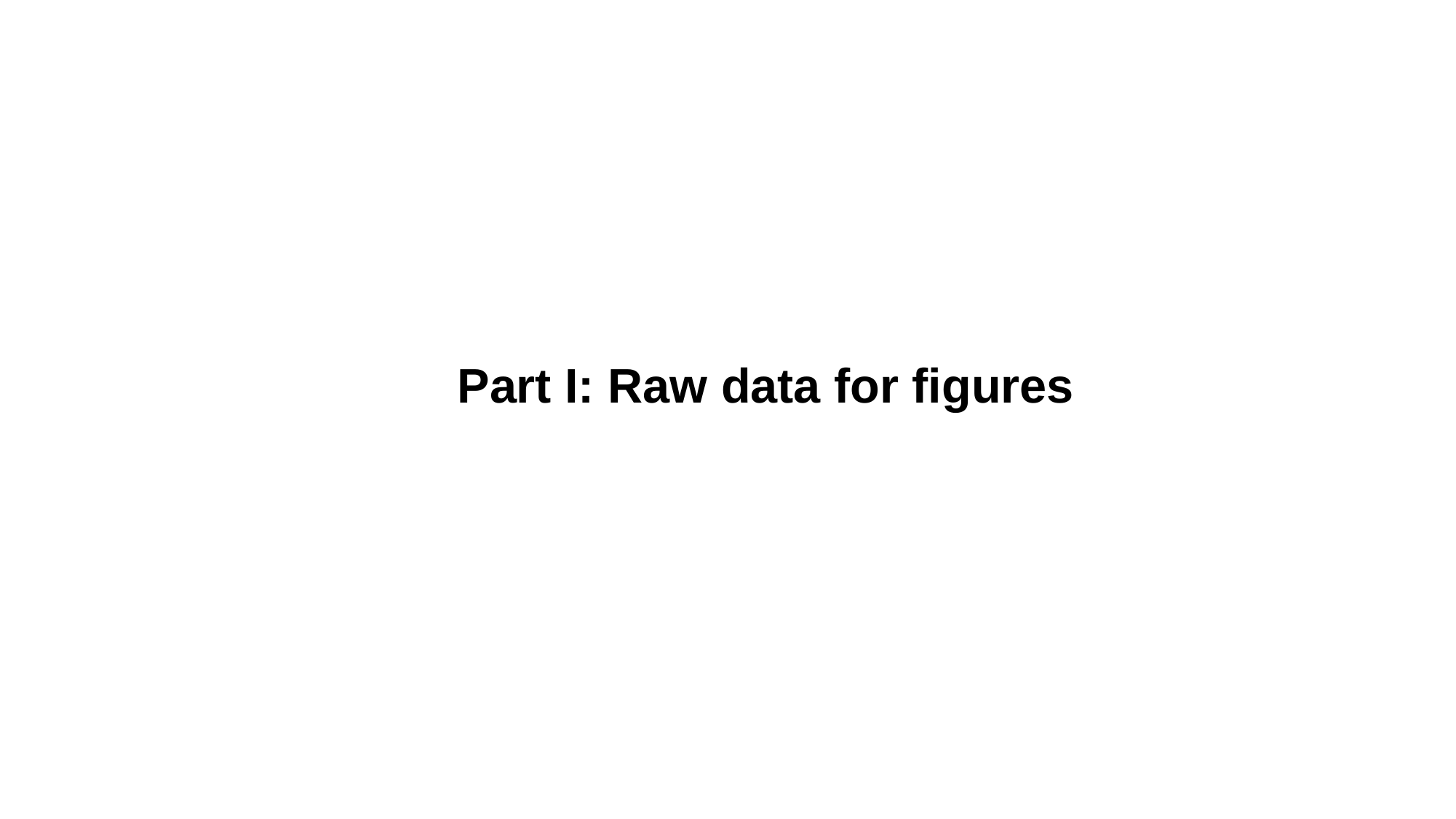

Part I: Raw data for figures

## Slide 2
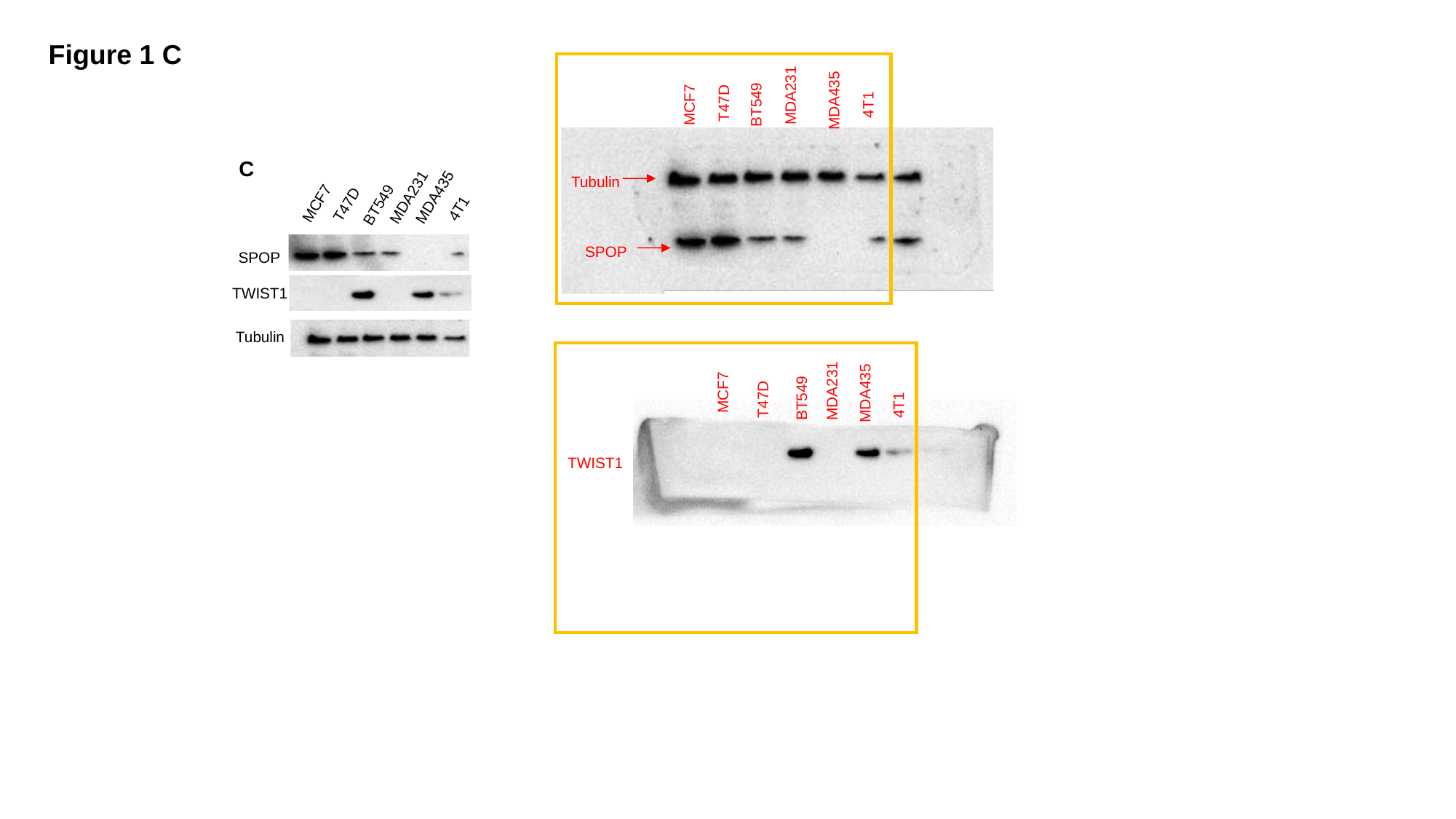

Figure 1 C
 MDA231
 MDA435
 T47D
 BT549
 4T1
 MCF7
C
 MDA231
 MDA435
 MCF7
 T47D
 BT549
 4T1
SPOP
TWIST1
Tubulin
Tubulin
SPOP
 MDA231
 MCF7
 MDA435
 BT549
 T47D
 4T1
TWIST1

## Slide 3
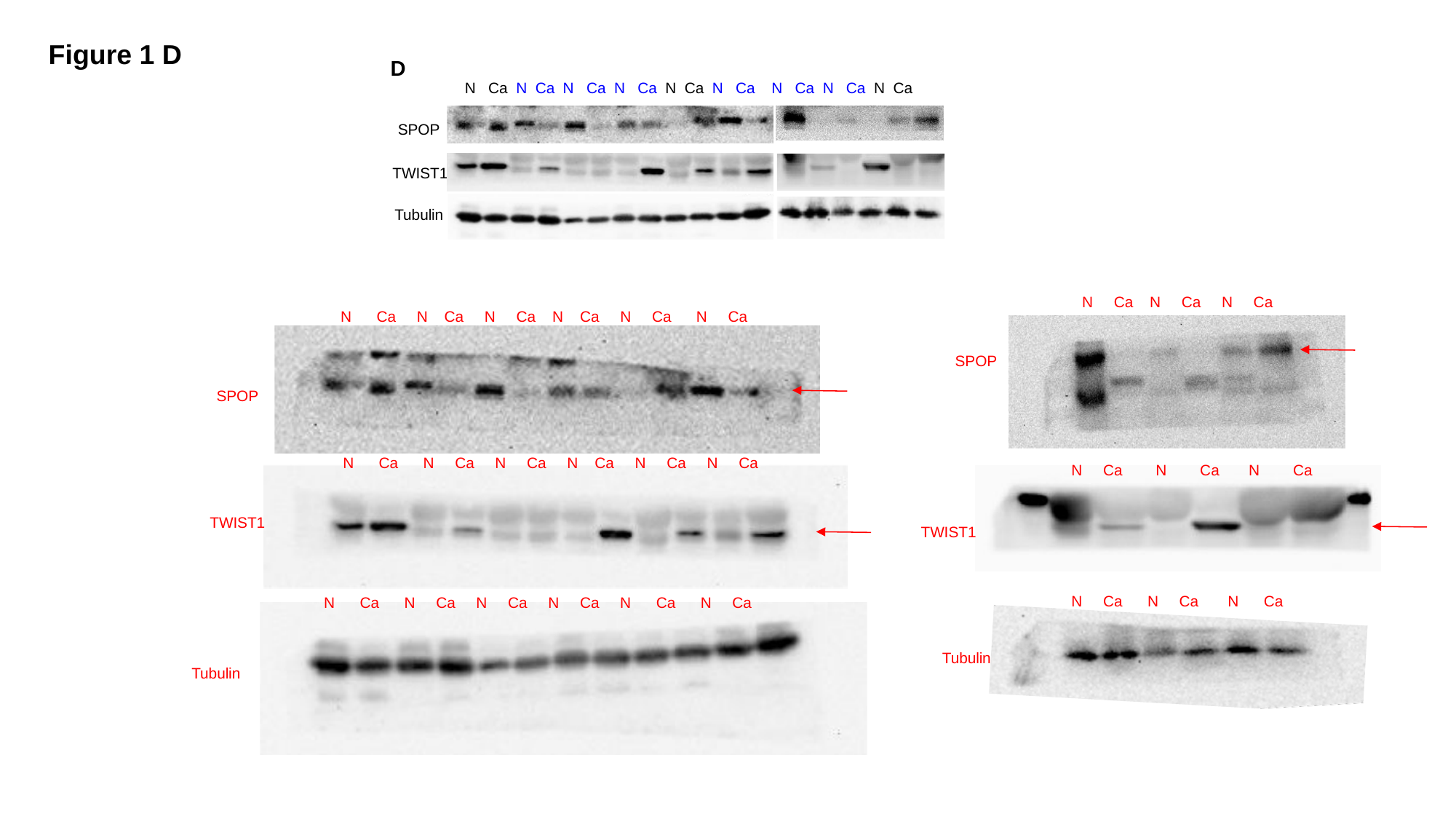

Figure 1 D
D
 N Ca N Ca N Ca N Ca N Ca N Ca N Ca N Ca N Ca
SPOP
TWIST1
Tubulin
N Ca N Ca N Ca
N Ca N Ca N Ca N Ca N Ca N Ca
SPOP
SPOP
N Ca N Ca N Ca N Ca N Ca N Ca
N Ca N Ca N Ca
TWIST1
TWIST1
N Ca N Ca N Ca
N Ca N Ca N Ca N Ca N Ca N Ca
Tubulin
Tubulin

## Slide 4
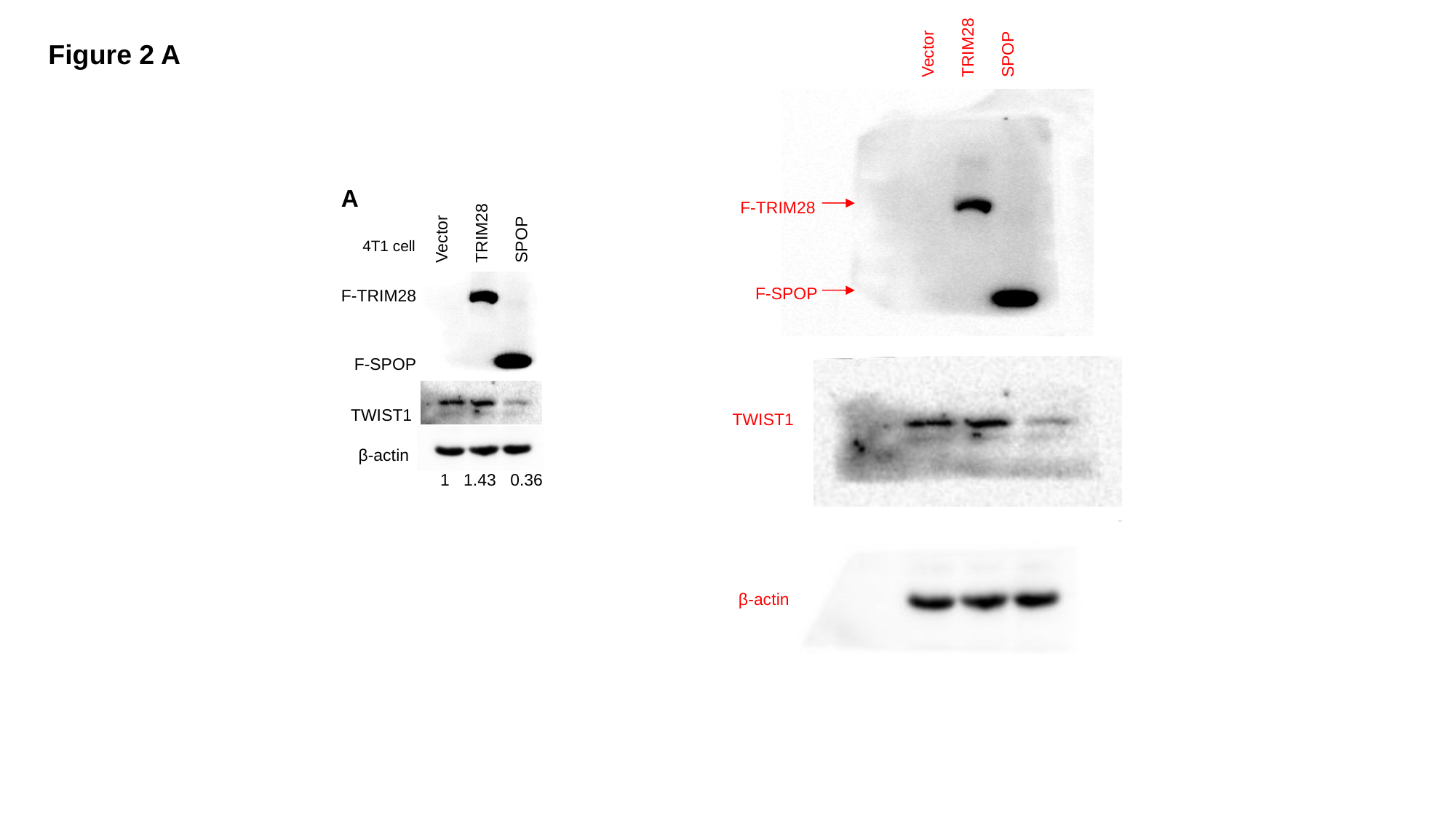

Vector
TRIM28
SPOP
Figure 2 A
Vector
TRIM28
SPOP
A
4T1 cell
F-TRIM28
F-SPOP
TWIST1
β-actin
1 1.43 0.36
F-TRIM28
F-SPOP
TWIST1
β-actin

## Slide 5
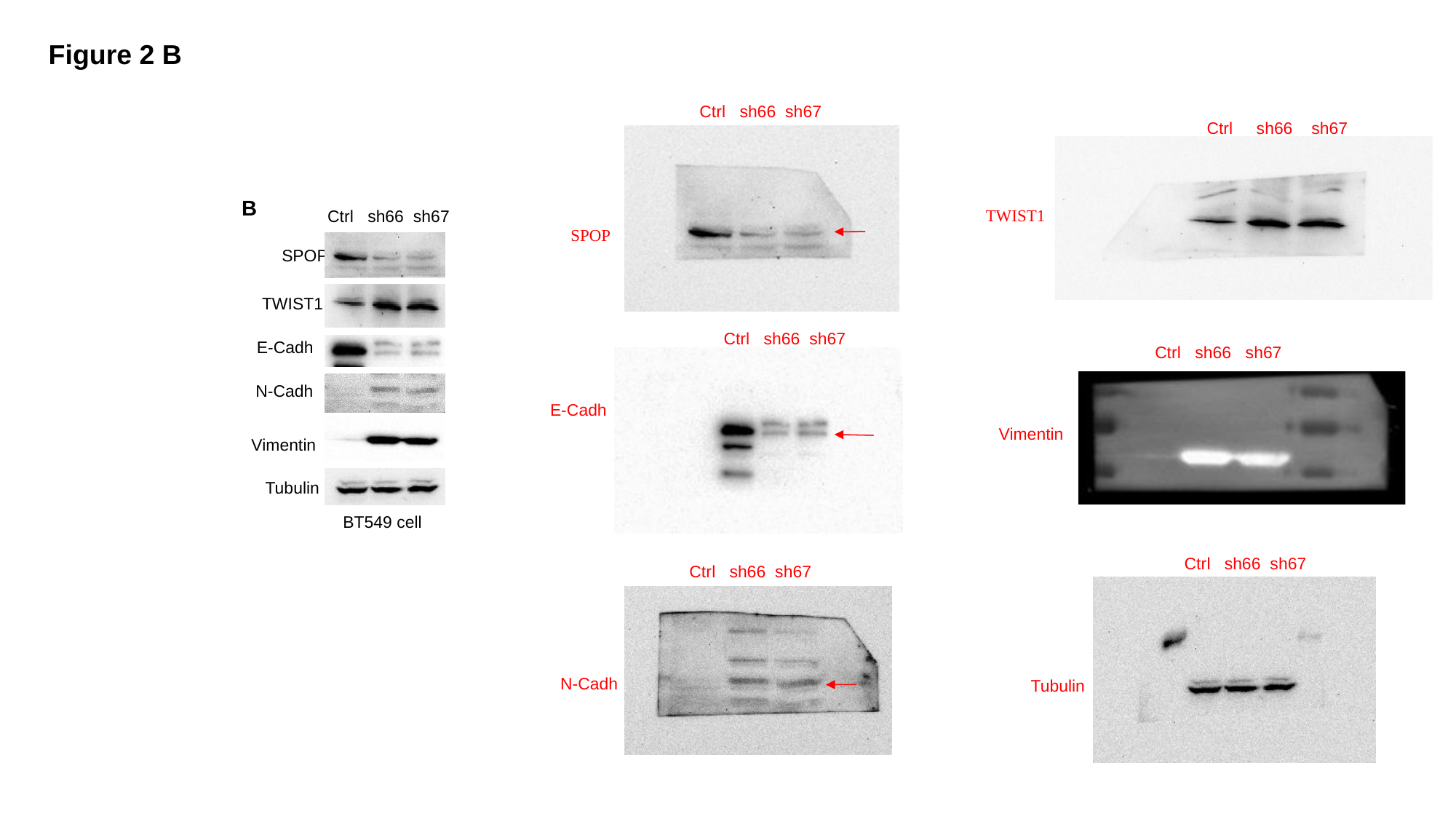

Figure 2 B
Ctrl sh66 sh67
Ctrl sh66 sh67
B
Ctrl sh66 sh67
SPOP
TWIST1
E-Cadh
N-Cadh
Vimentin
Tubulin
BT549 cell
TWIST1
SPOP
Ctrl sh66 sh67
Ctrl sh66 sh67
E-Cadh
Vimentin
Ctrl sh66 sh67
Ctrl sh66 sh67
N-Cadh
Tubulin

## Slide 6
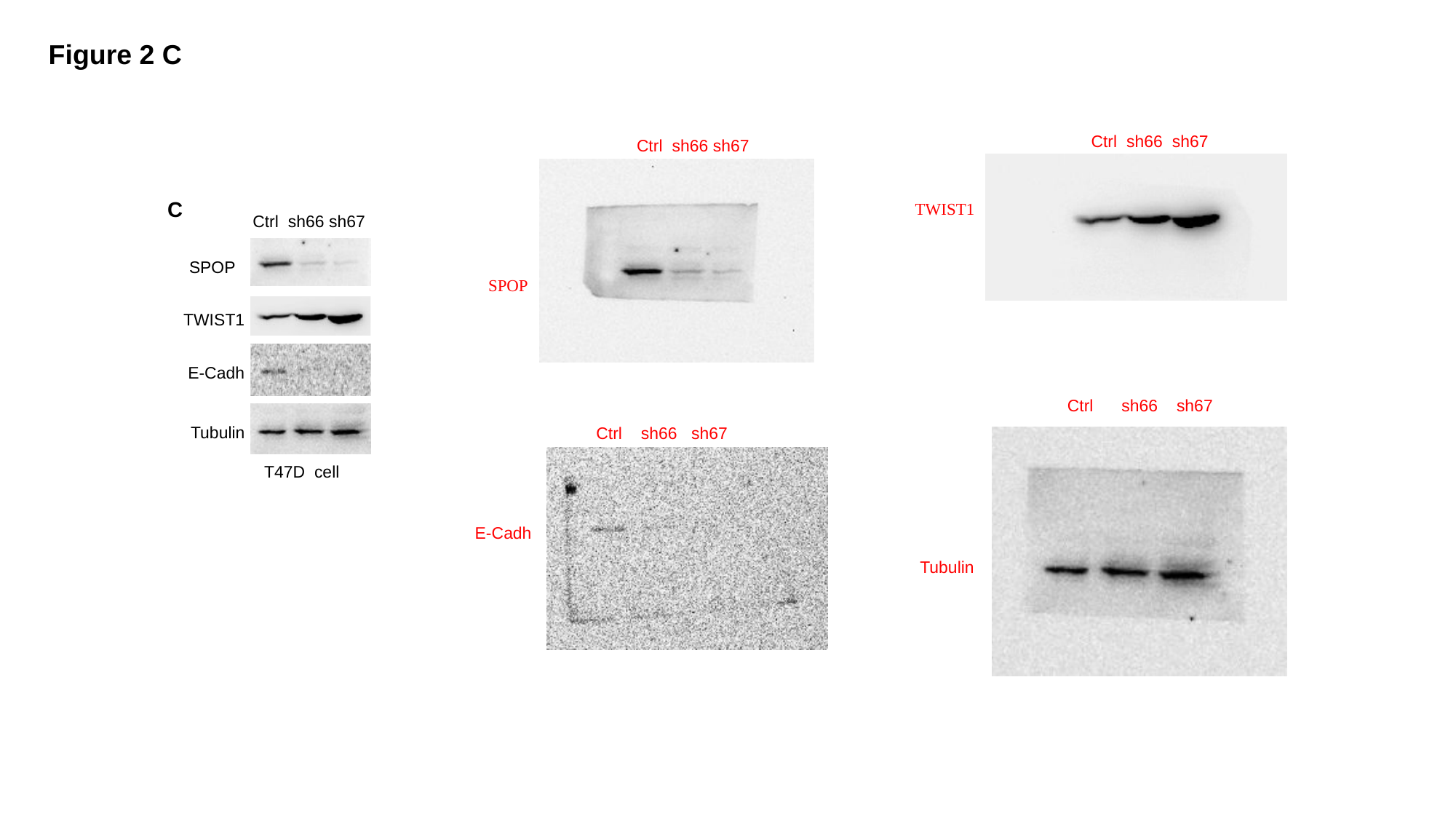

Figure 2 C
Ctrl sh66 sh67
Ctrl sh66 sh67
C
Ctrl sh66 sh67
SPOP
TWIST1
E-Cadh
Tubulin
T47D cell
TWIST1
SPOP
Ctrl sh66 sh67
Ctrl sh66 sh67
E-Cadh
Tubulin

## Slide 7
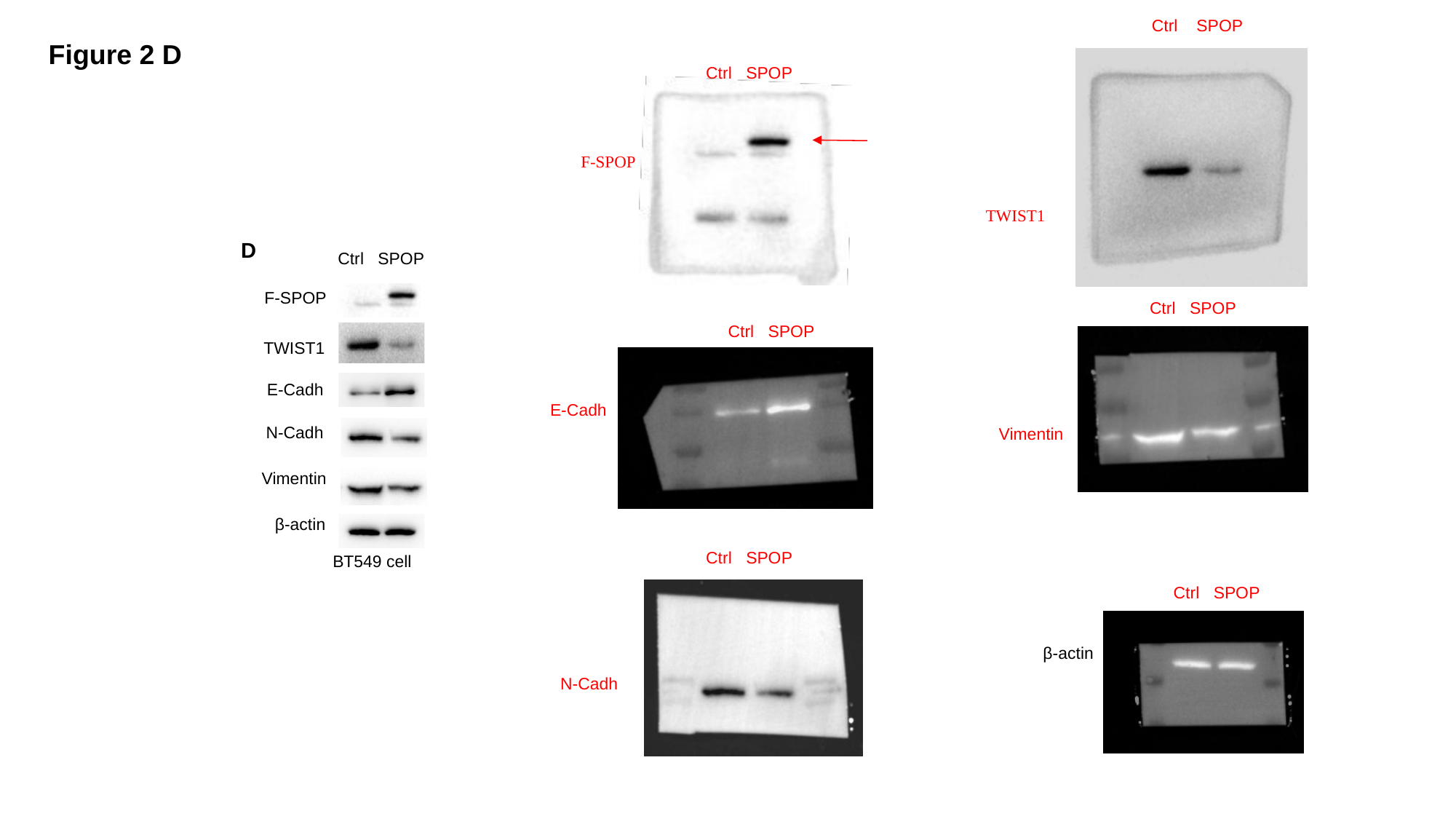

Ctrl SPOP
Figure 2 D
Ctrl SPOP
F-SPOP
TWIST1
D
Ctrl SPOP
F-SPOP
TWIST1
E-Cadh
N-Cadh
Vimentin
β-actin
BT549 cell
Ctrl SPOP
Ctrl SPOP
E-Cadh
Vimentin
Ctrl SPOP
Ctrl SPOP
β-actin
N-Cadh

## Slide 8
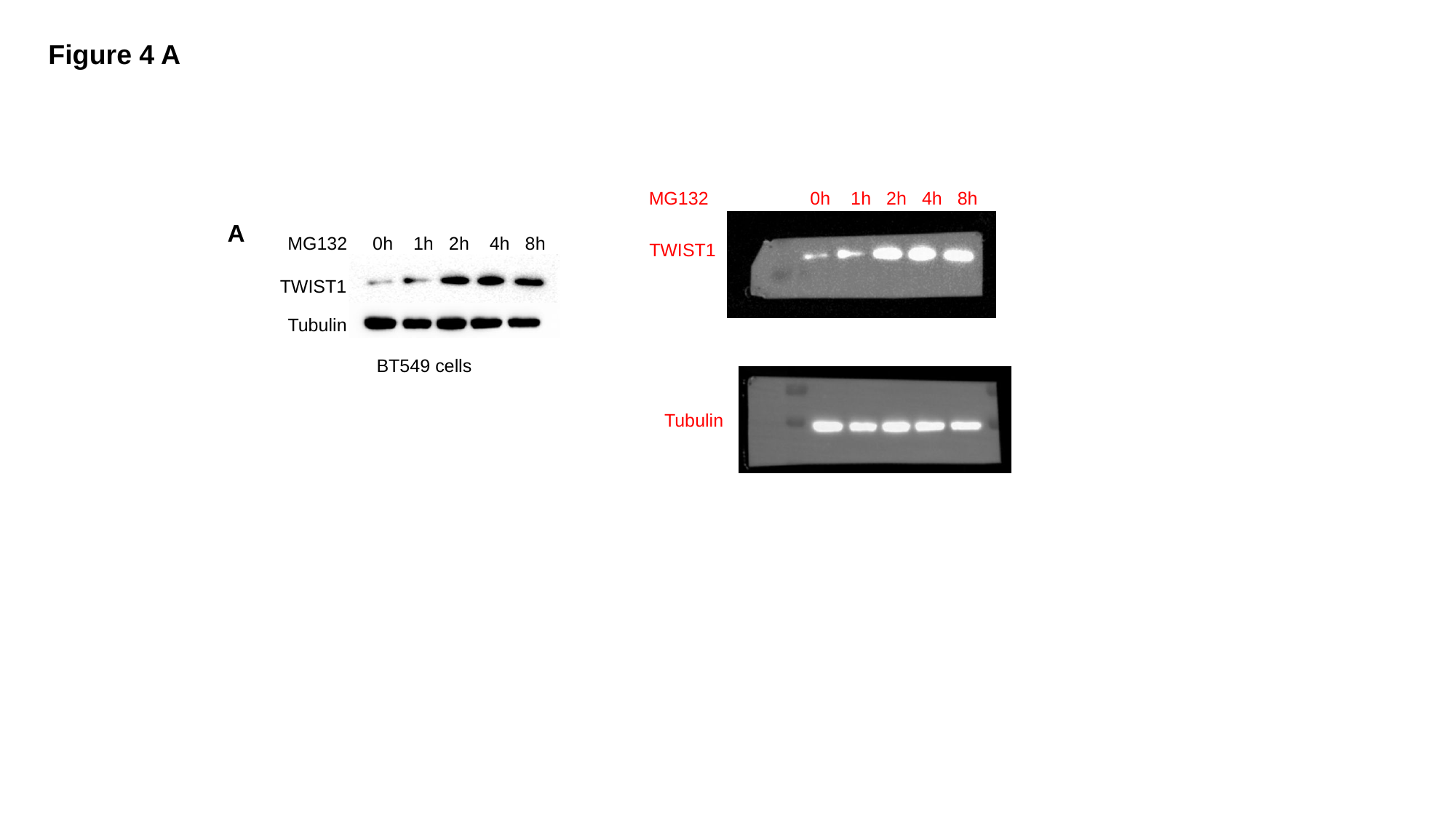

Figure 4 A
MG132 0h 1h 2h 4h 8h
TWIST1
Tubulin
A
MG132 0h 1h 2h 4h 8h
TWIST1
Tubulin
BT549 cells

## Slide 9
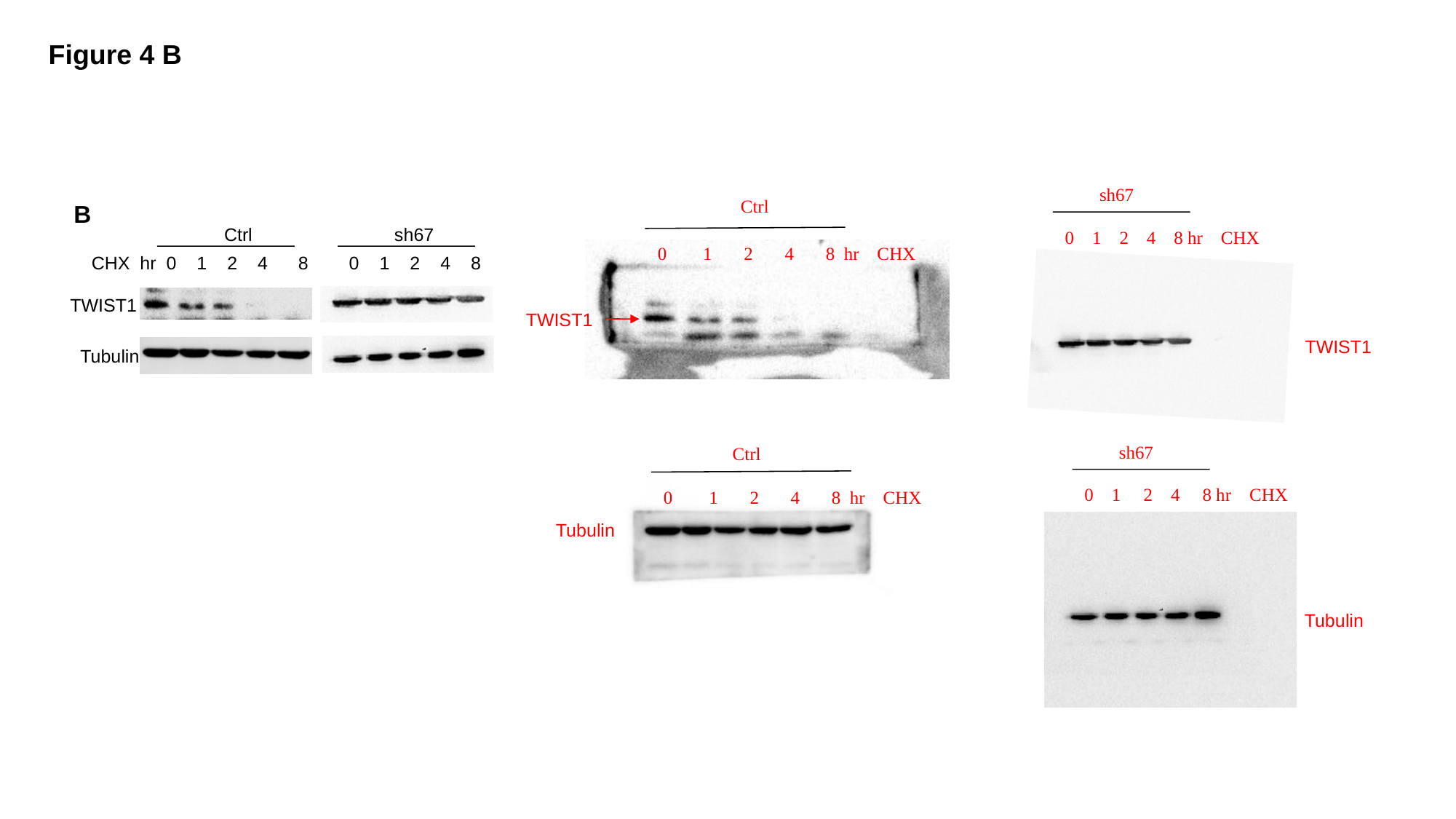

Figure 4 B
sh67
Ctrl
B
 Ctrl sh67
 CHX hr 0 1 2 4 8 0 1 2 4 8
TWIST1
Tubulin
0 1 2 4 8 hr CHX
0 1 2 4 8 hr CHX
TWIST1
TWIST1
sh67
Ctrl
0 1 2 4 8 hr CHX
0 1 2 4 8 hr CHX
Tubulin
Tubulin

## Slide 10
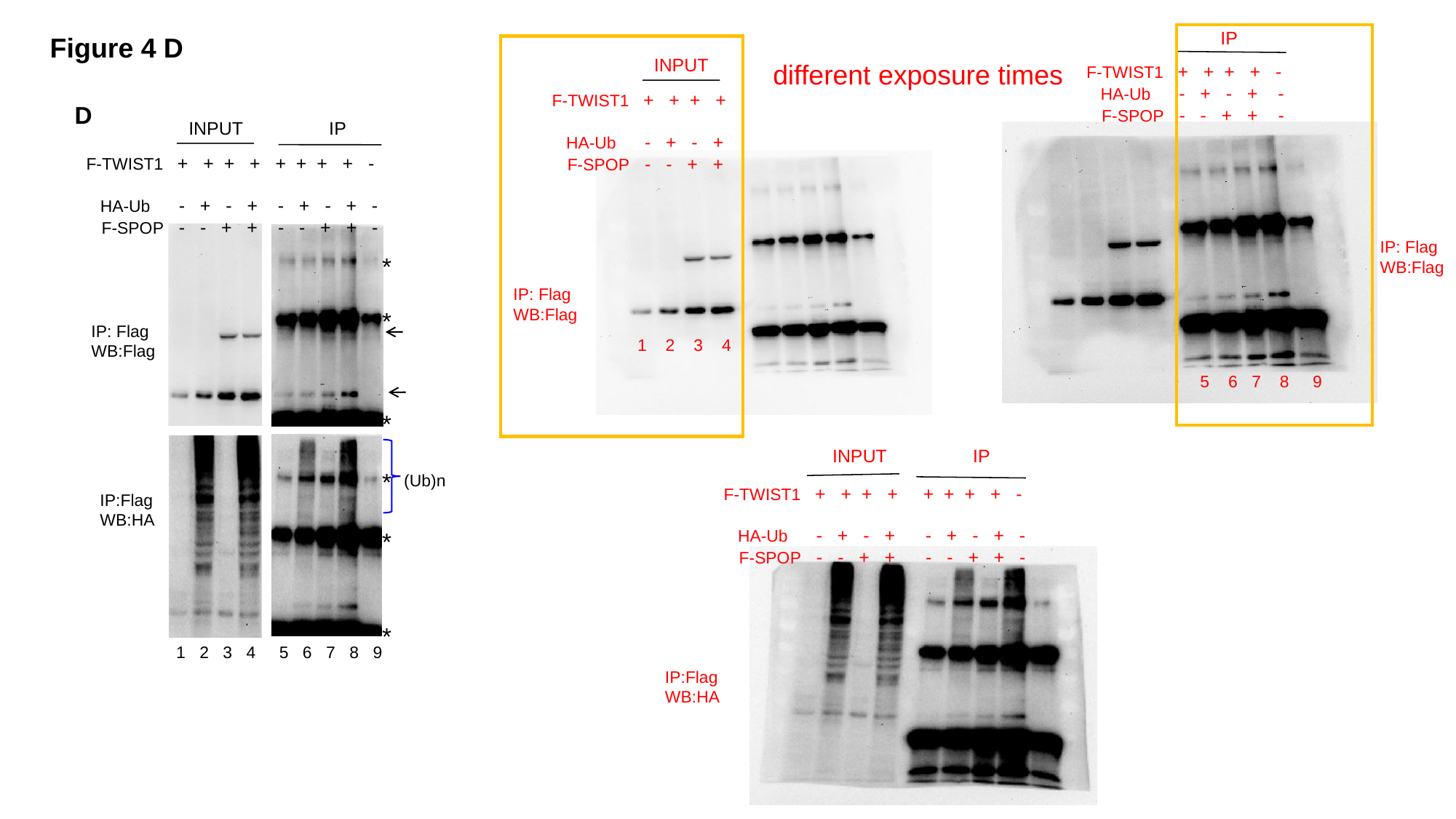

IP
Figure 4 D
INPUT
different exposure times
F-TWIST1 + + + + -
 HA-Ub - + - + -
 F-SPOP - - + + -
F-TWIST1 + + + +
 HA-Ub - + - +
 F-SPOP - - + +
D
INPUT IP
F-TWIST1 + + + + + + + + -
 HA-Ub - + - + - + - + -
 F-SPOP - - + + - - + + -
*
*
IP: Flag
WB:Flag
*
*
(Ub)n
IP:Flag
WB:HA
*
*
1 2 3 4 5 6 7 8 9
IP: Flag
WB:Flag
IP: Flag
WB:Flag
1 2 3 4
5 6 7 8 9
INPUT IP
F-TWIST1 + + + + + + + + -
 HA-Ub - + - + - + - + -
 F-SPOP - - + + - - + + -
IP:Flag
WB:HA

## Slide 11
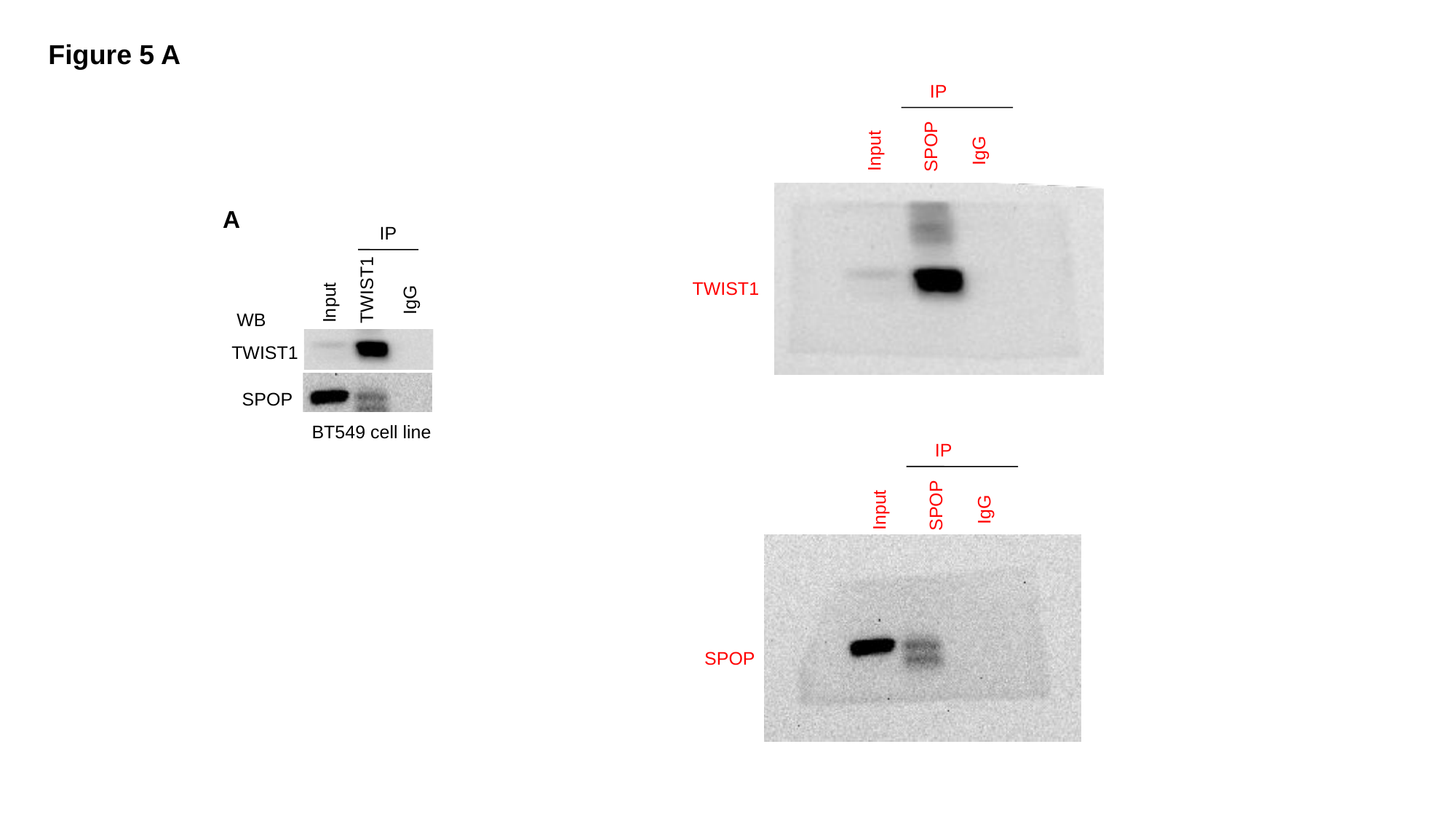

Figure 5 A
IP
SPOP
IgG
Input
TWIST1
A
IP
TWIST1
IgG
Input
 WB
TWIST1
SPOP
BT549 cell line
IP
SPOP
IgG
Input
SPOP

## Slide 12
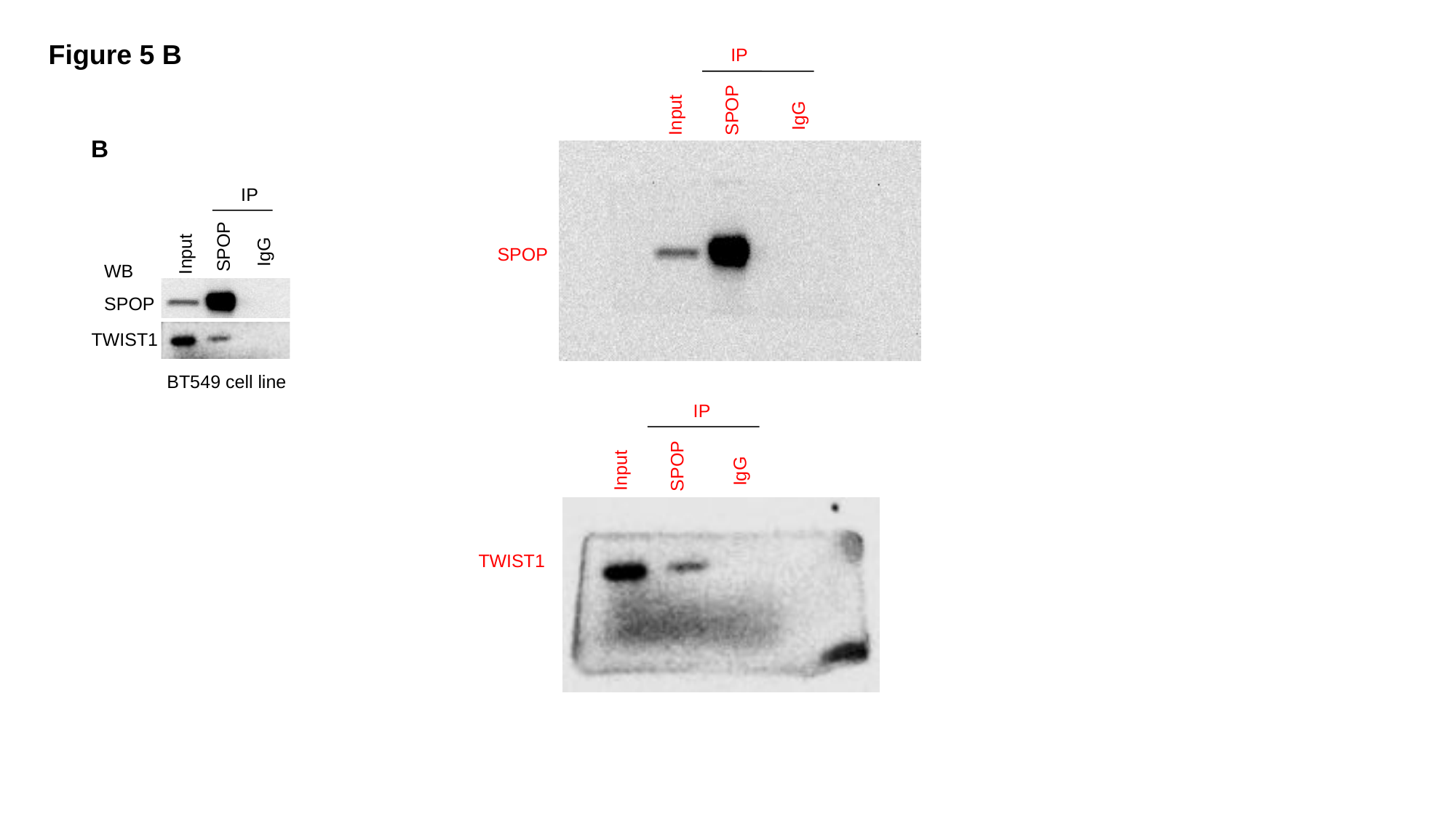

Figure 5 B
IP
SPOP
Input
IgG
SPOP
B
IP
SPOP
IgG
Input
WB
SPOP
TWIST1
BT549 cell line
IP
SPOP
Input
IgG
TWIST1

## Slide 13
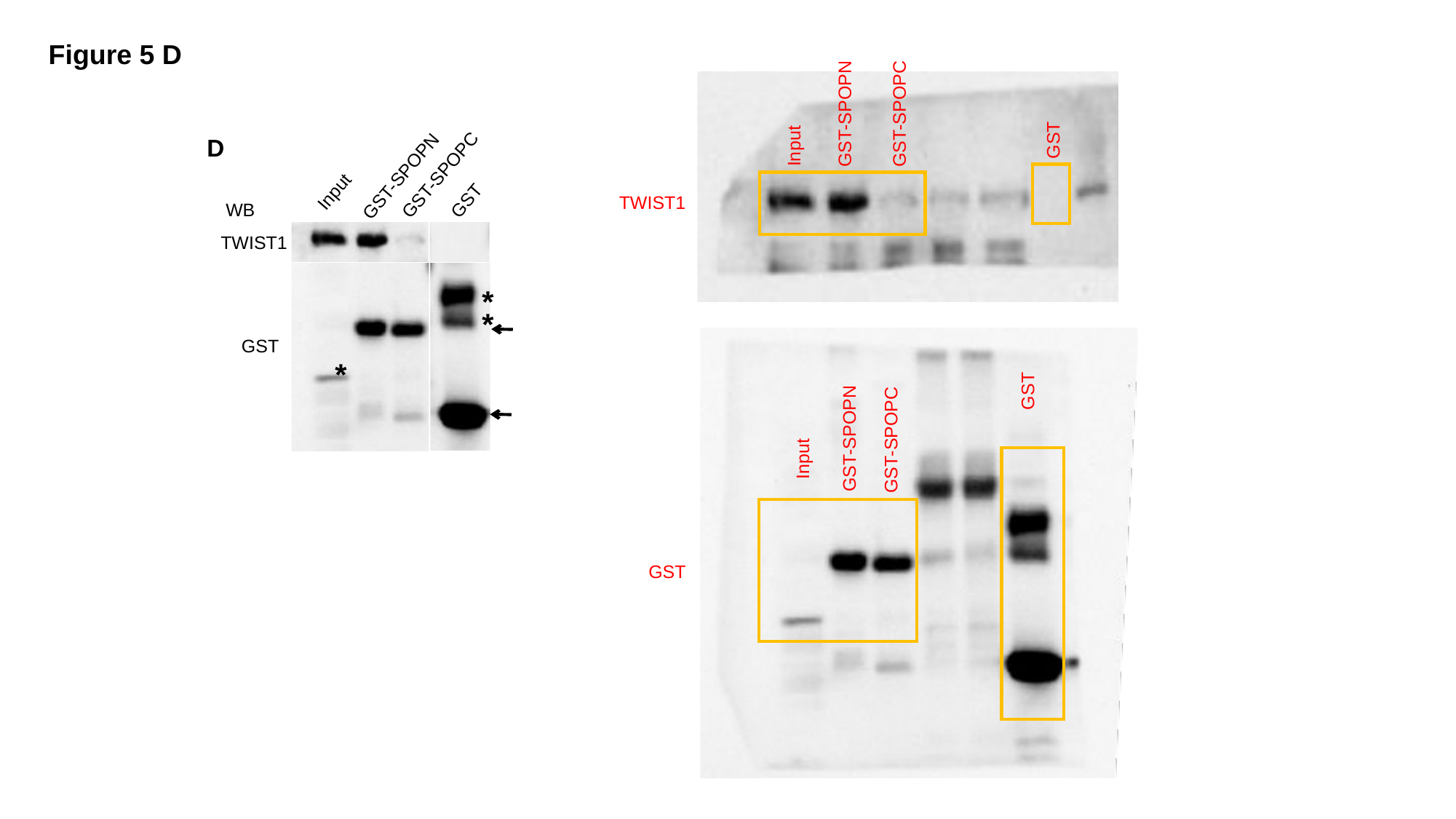

Figure 5 D
GST-SPOPN
GST-SPOPC
D
GST-SPOPC
GST-SPOPN
Input
 WB
TWIST1
GST
*
*
GST
*
GST
Input
TWIST1
GST
GST-SPOPN
GST-SPOPC
Input
GST

## Slide 14
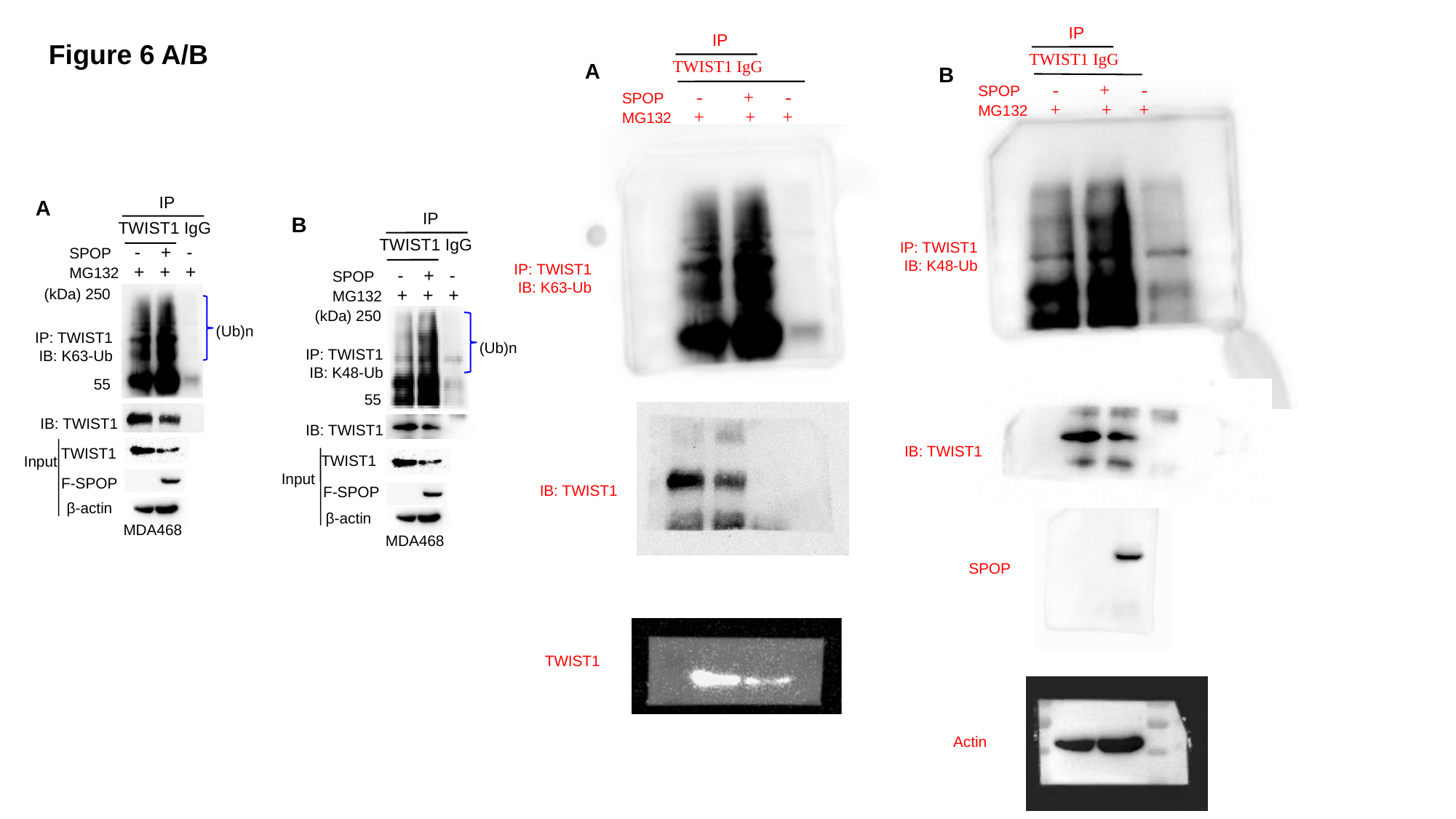

IP
TWIST1 IgG
SPOP - + -
MG132 + + +
IP
Figure 6 A/B
TWIST1 IgG
A
B
SPOP - + -
MG132 + + +
IP
A
TWIST1 IgG
SPOP - + -
MG132 + + +
(kDa) 250
55
(Ub)n
IP: TWIST1
IB: K63-Ub
IB: TWIST1
TWIST1
Input
F-SPOP
β-actin
MDA468
IP
B
TWIST1 IgG
SPOP - + -
MG132 + + +
(kDa) 250
55
(Ub)n
IP: TWIST1
IB: K48-Ub
IB: TWIST1
TWIST1
Input
F-SPOP
β-actin
MDA468
IP: TWIST1
IB: K48-Ub
IP: TWIST1
IB: K63-Ub
IB: TWIST1
IB: TWIST1
SPOP
TWIST1
Actin

## Slide 15
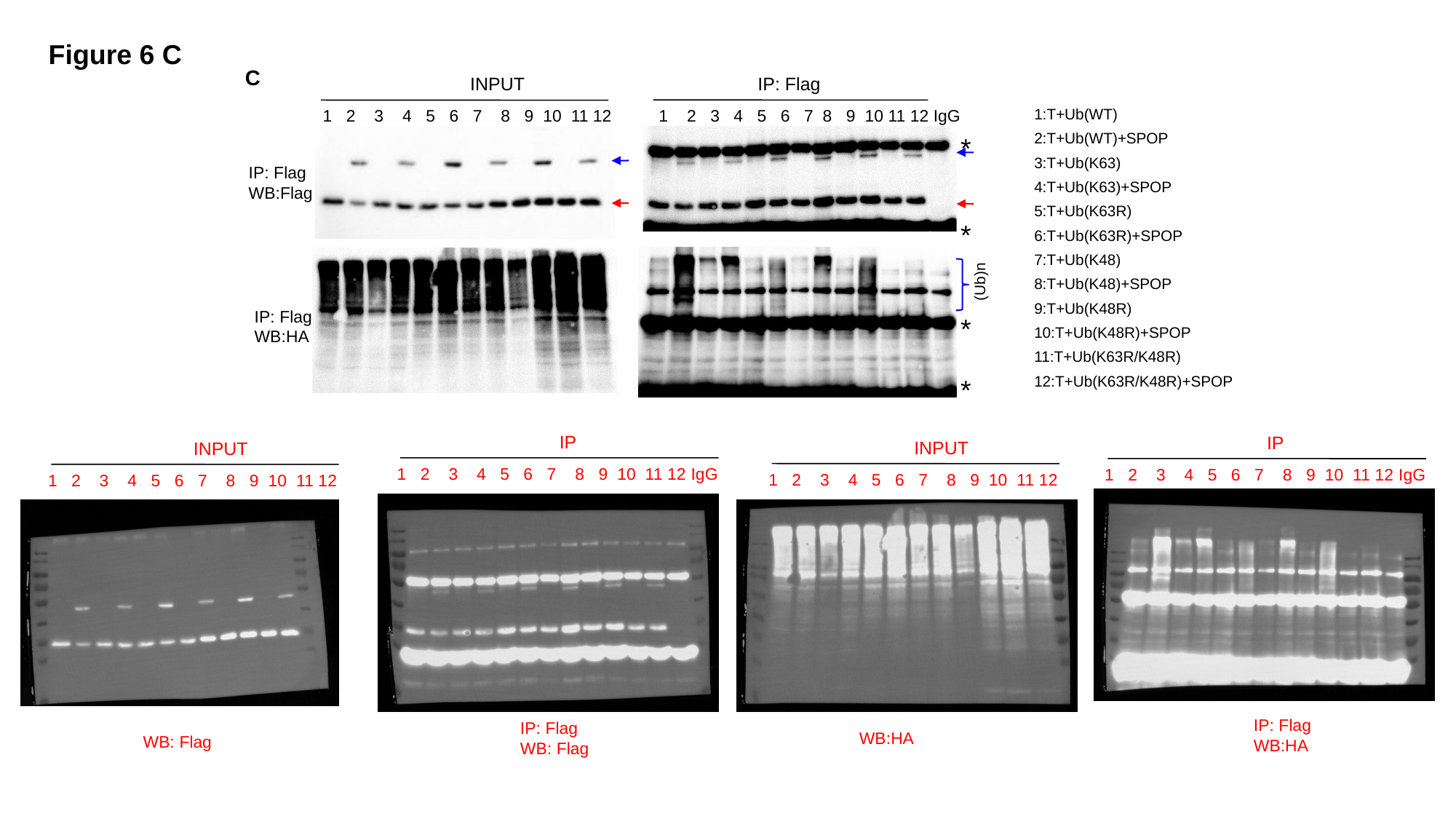

Figure 6 C
C
INPUT IP: Flag
1 2 3 4 5 6 7 8 9 10 11 12 1 2 3 4 5 6 7 8 9 10 11 12 IgG
*
IP: Flag
WB:Flag
*
(Ub)n
IP: Flag
WB:HA
*
*
1:T+Ub(WT)
2:T+Ub(WT)+SPOP
3:T+Ub(K63)
4:T+Ub(K63)+SPOP
5:T+Ub(K63R)
6:T+Ub(K63R)+SPOP
7:T+Ub(K48)
8:T+Ub(K48)+SPOP
9:T+Ub(K48R)
10:T+Ub(K48R)+SPOP
11:T+Ub(K63R/K48R)
12:T+Ub(K63R/K48R)+SPOP
IP
IP
INPUT
INPUT
1 2 3 4 5 6 7 8 9 10 11 12 IgG
1 2 3 4 5 6 7 8 9 10 11 12 IgG
1 2 3 4 5 6 7 8 9 10 11 12
1 2 3 4 5 6 7 8 9 10 11 12
IP: Flag
WB:HA
IP: Flag
WB: Flag
WB:HA
WB: Flag

## Slide 16
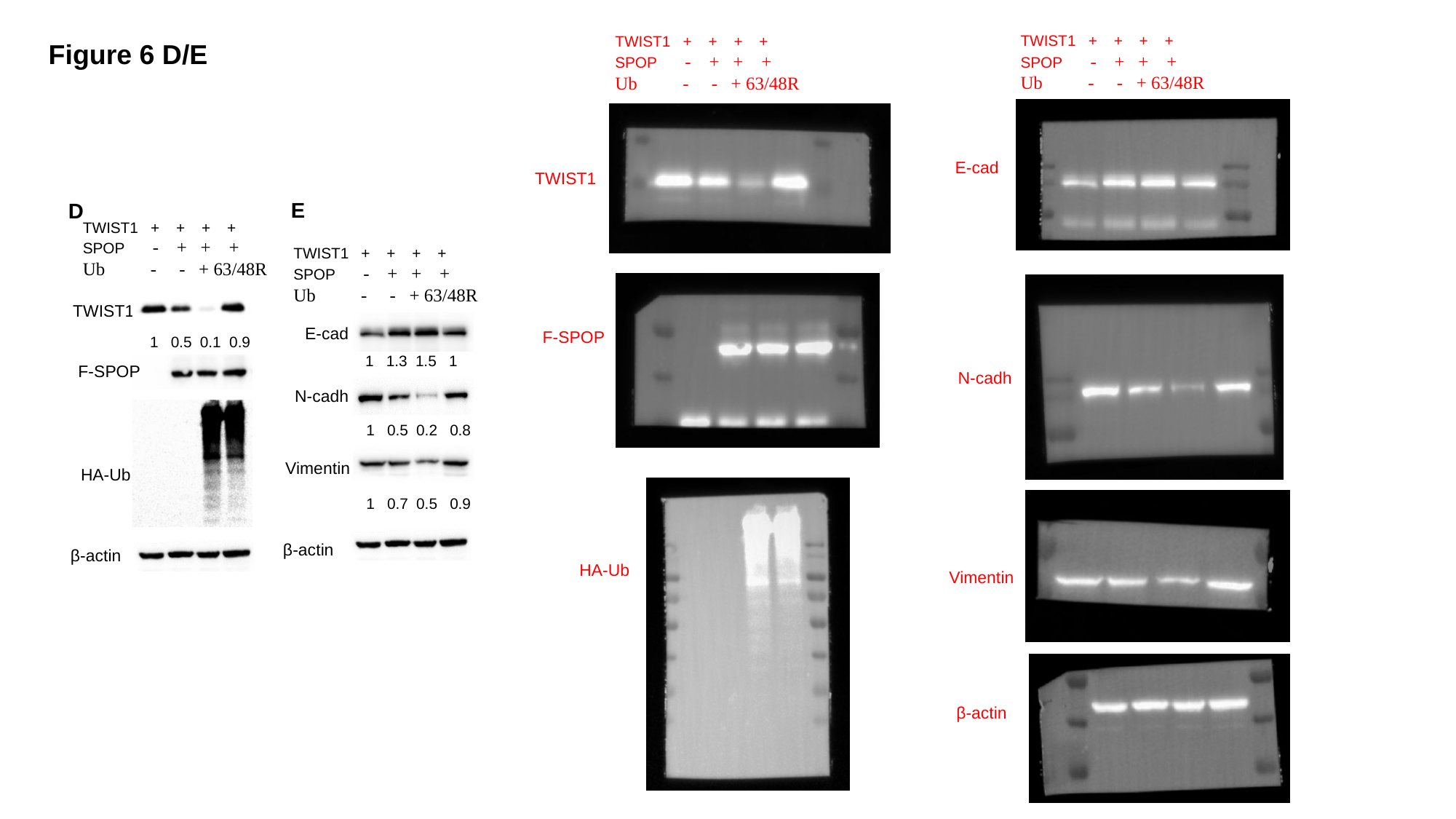

TWIST1 + + + +
SPOP - + + +
Ub - - + 63/48R
TWIST1 + + + +
SPOP - + + +
Ub - - + 63/48R
Figure 6 D/E
E-cad
TWIST1
E
D
TWIST1 + + + +
SPOP - + + +
Ub - - + 63/48R
TWIST1 + + + +
SPOP - + + +
Ub - - + 63/48R
TWIST1
E-cad
1 0.5 0.1 0.9
1 1.3 1.5 1
F-SPOP
N-cadh
1 0.5 0.2 0.8
Vimentin
HA-Ub
1 0.7 0.5 0.9
β-actin
β-actin
F-SPOP
N-cadh
HA-Ub
Vimentin
β-actin

## Slide 17
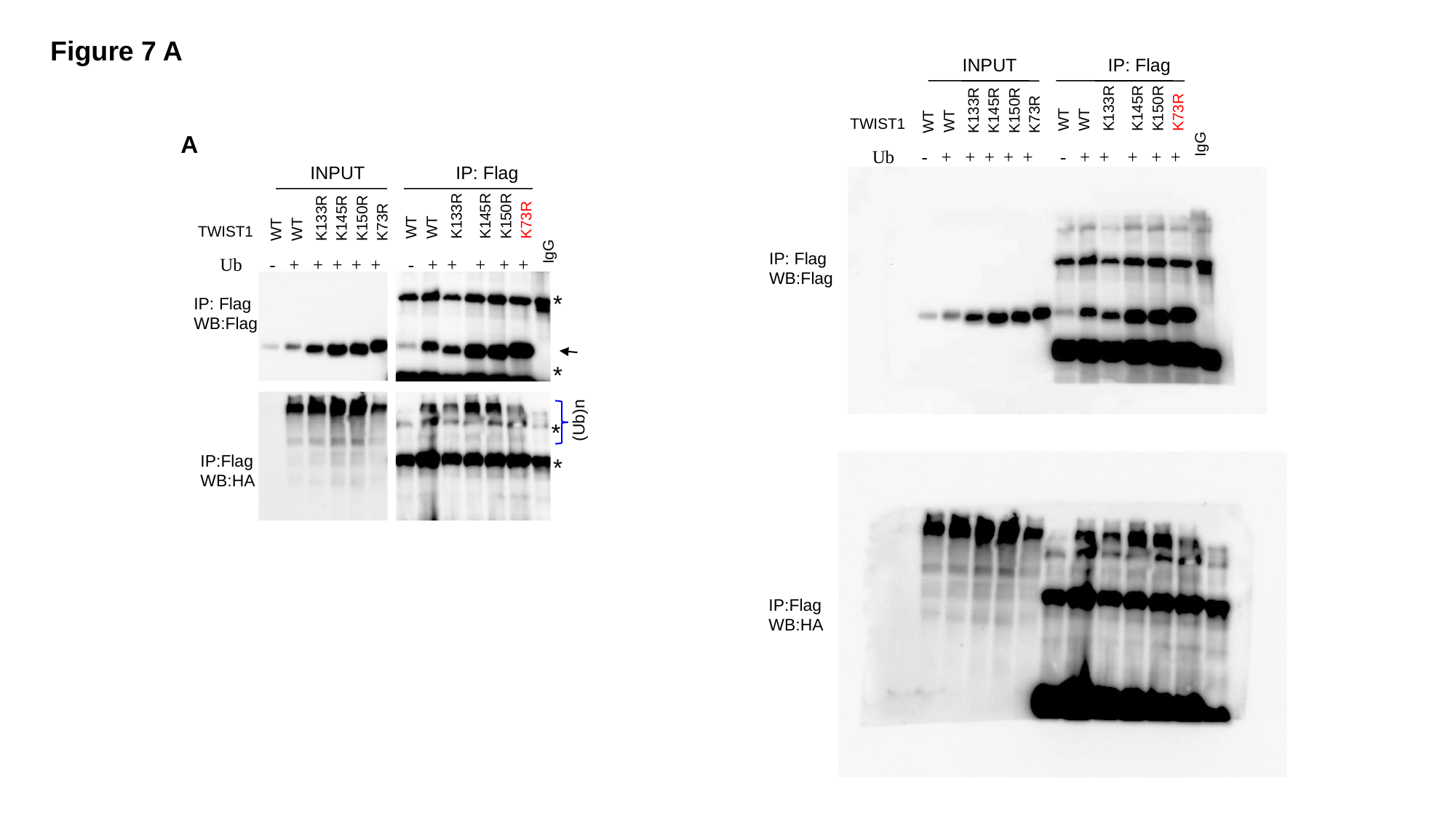

Figure 7 A
INPUT IP: Flag
K145R
K145R
K133R
K150R
K133R
K150R
K73R
K73R
WT
WT
WT
WT
TWIST1
IgG
 Ub - + + + + + - + + + + +
A
INPUT IP: Flag
K145R
K145R
K133R
K150R
K133R
K150R
K73R
K73R
WT
WT
WT
WT
TWIST1
IgG
 Ub - + + + + + - + + + + +
*
IP: Flag
WB:Flag
*
(Ub)n
*
IP:Flag
WB:HA
*
IP: Flag
WB:Flag
IP:Flag
WB:HA

## Slide 18
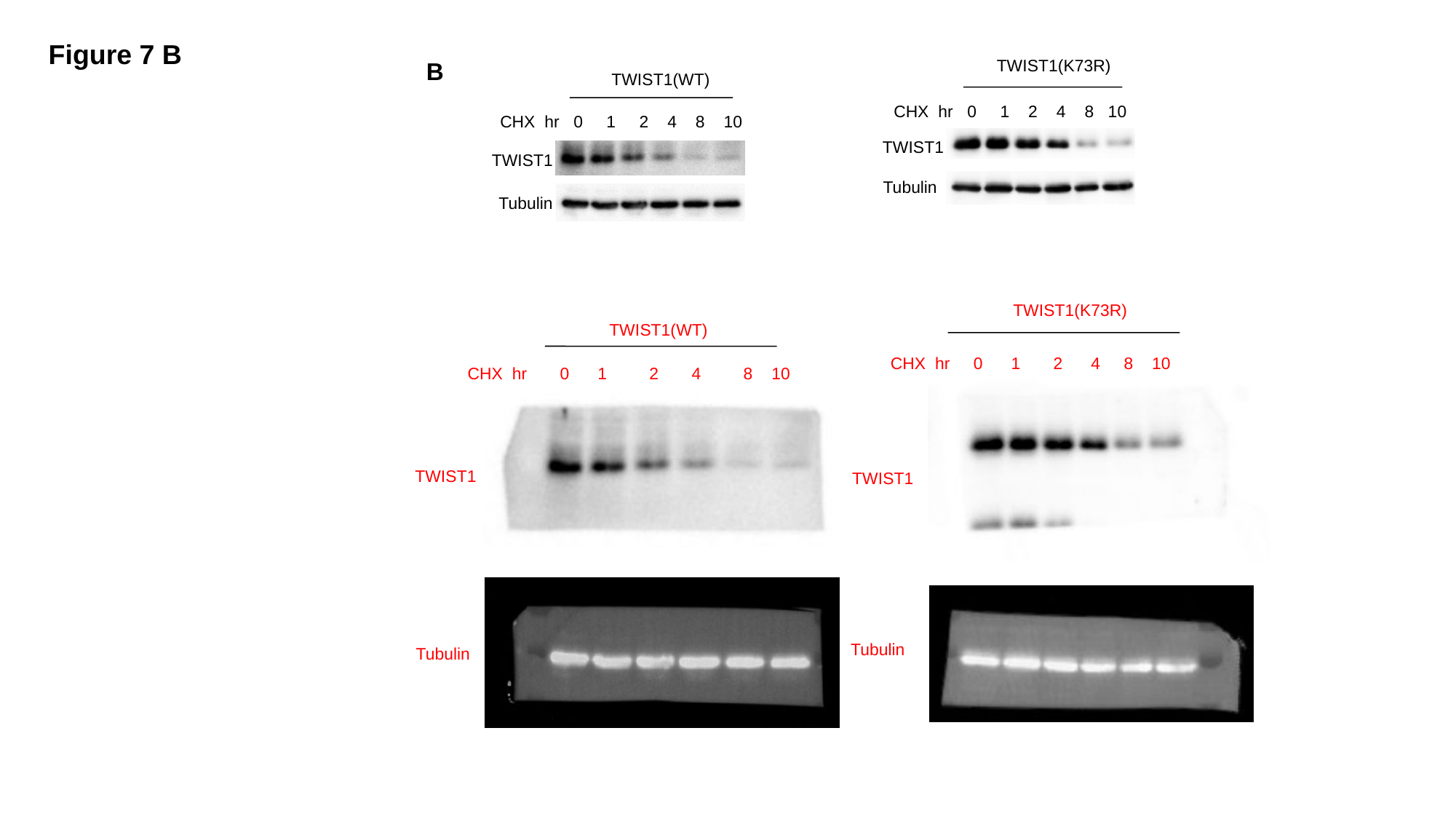

Figure 7 B
TWIST1(K73R)
 CHX hr 0 1 2 4 8 10
TWIST1
Tubulin
B
TWIST1(WT)
 CHX hr 0 1 2 4 8 10
TWIST1
Tubulin
TWIST1(K73R)
TWIST1(WT)
 CHX hr 0 1 2 4 8 10
 CHX hr 0 1 2 4 8 10
TWIST1
TWIST1
Tubulin
Tubulin

## Slide 19
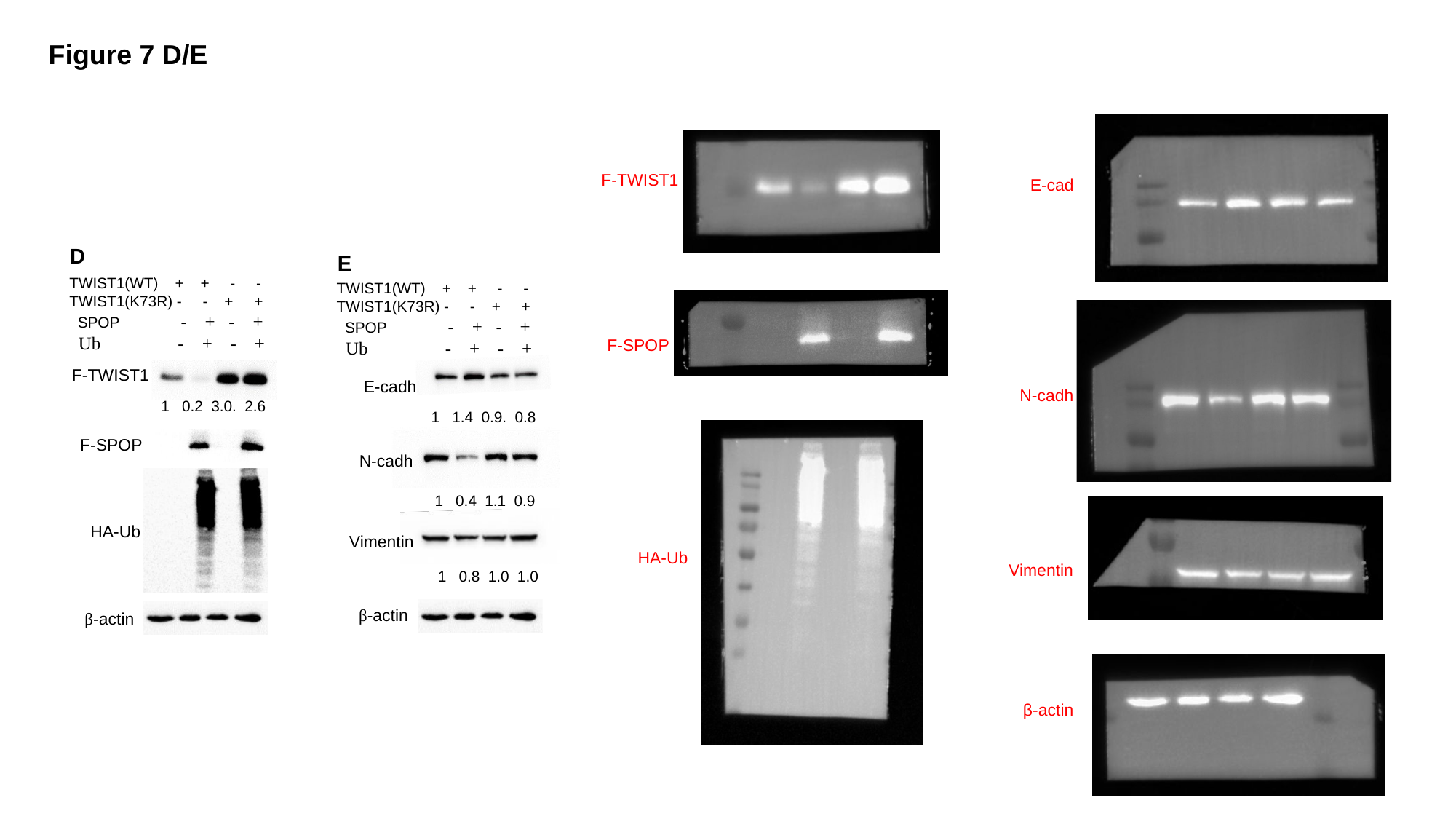

Figure 7 D/E
F-TWIST1
E-cad
D
TWIST1(WT) + + - -
TWIST1(K73R) - - + +
 SPOP - + - +
 Ub - + - +
F-TWIST1
1 0.2 3.0. 2.6
F-SPOP
HA-Ub
β-actin
E
TWIST1(WT) + + - -
TWIST1(K73R) - - + +
 SPOP - + - +
 Ub - + - +
E-cadh
1 1.4 0.9. 0.8
N-cadh
1 0.4 1.1 0.9
Vimentin
1 0.8 1.0 1.0
β-actin
F-SPOP
N-cadh
HA-Ub
Vimentin
β-actin

## Slide 20
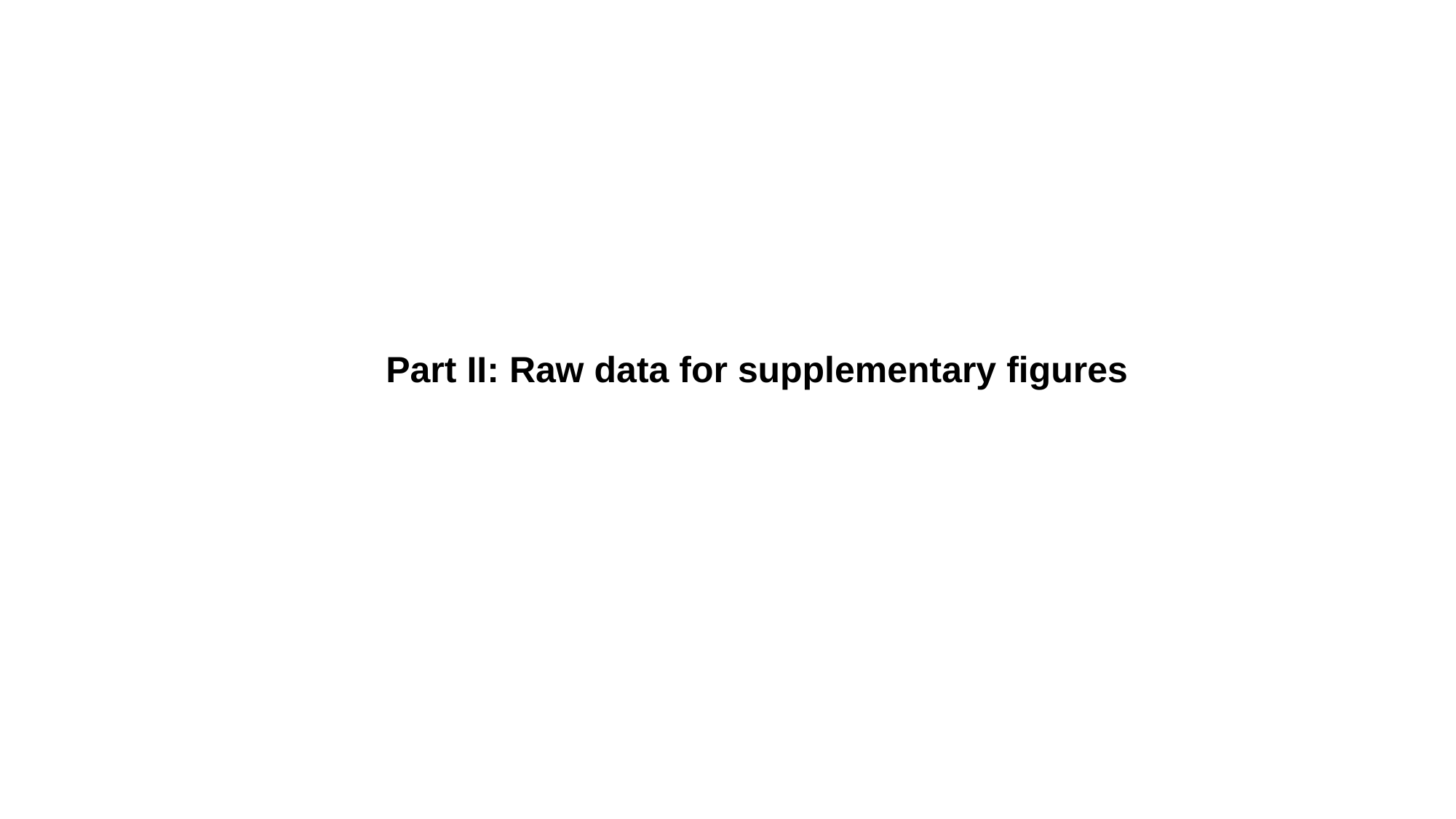

Part II: Raw data for supplementary figures

## Slide 21
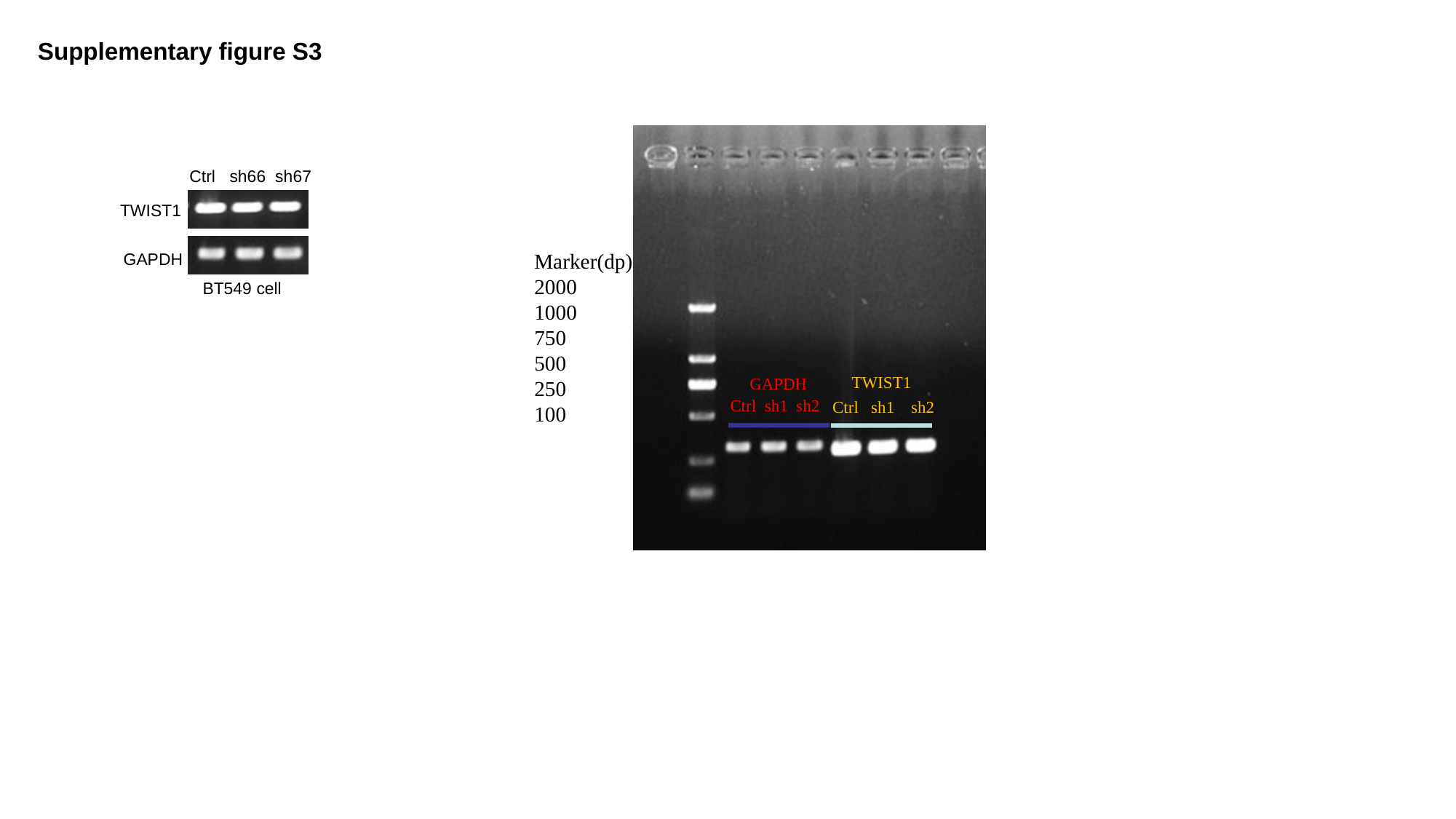

Supplementary figure S3
TWIST1
GAPDH
Ctrl sh1 sh2
Ctrl sh1 sh2
Ctrl sh66 sh67
TWIST1
GAPDH
BT549 cell
Marker(dp)
2000
1000
750
500
250
100

## Slide 22
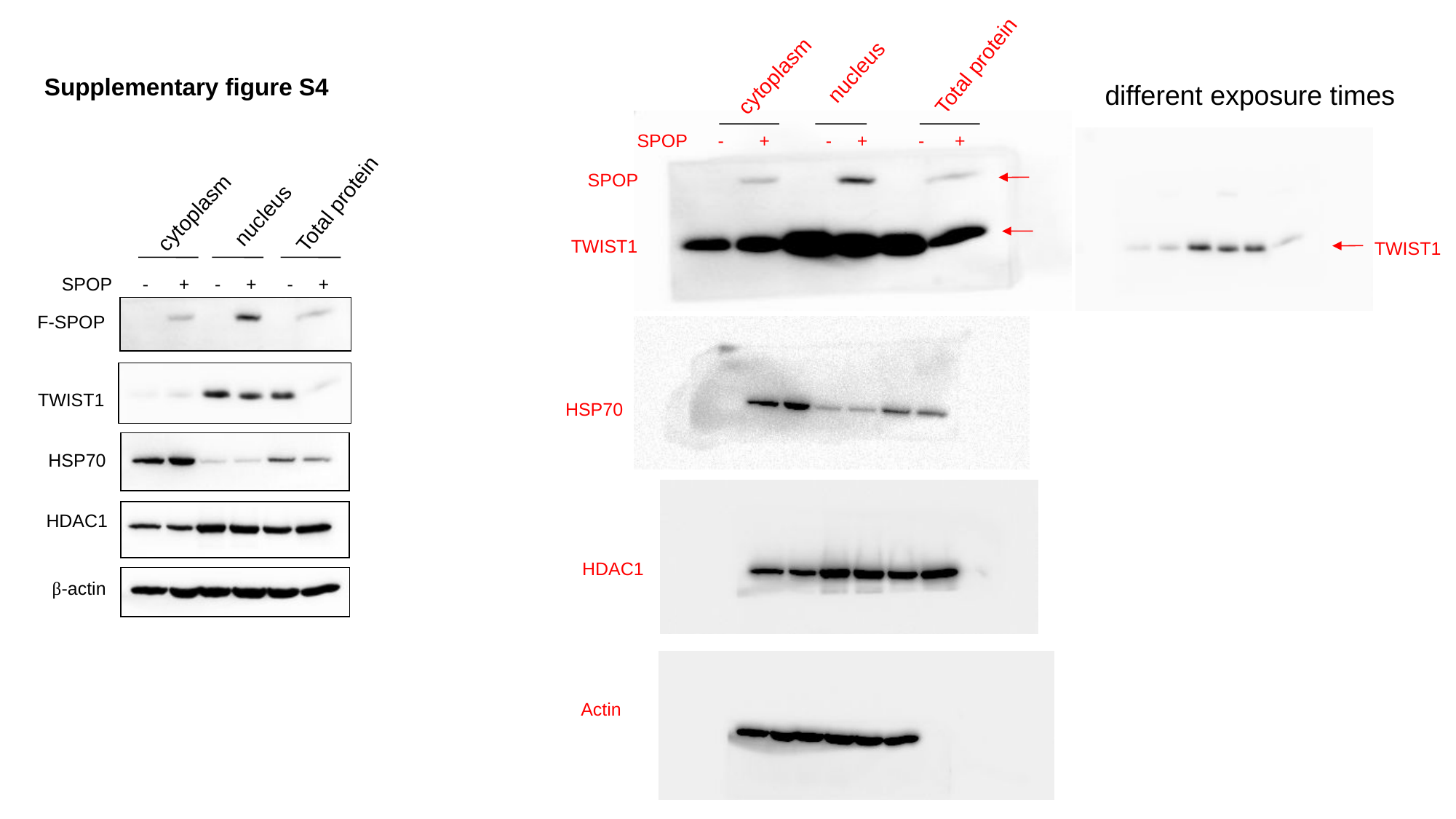

Total protein
nucleus
cytoplasm
Supplementary figure S4
different exposure times
SPOP - + - + - +
Total protein
cytoplasm
nucleus
SPOP - + - + - +
F-SPOP
TWIST1
HSP70
HDAC1
SPOP
TWIST1
TWIST1
HSP70
HDAC1
β-actin
Actin

## Slide 23
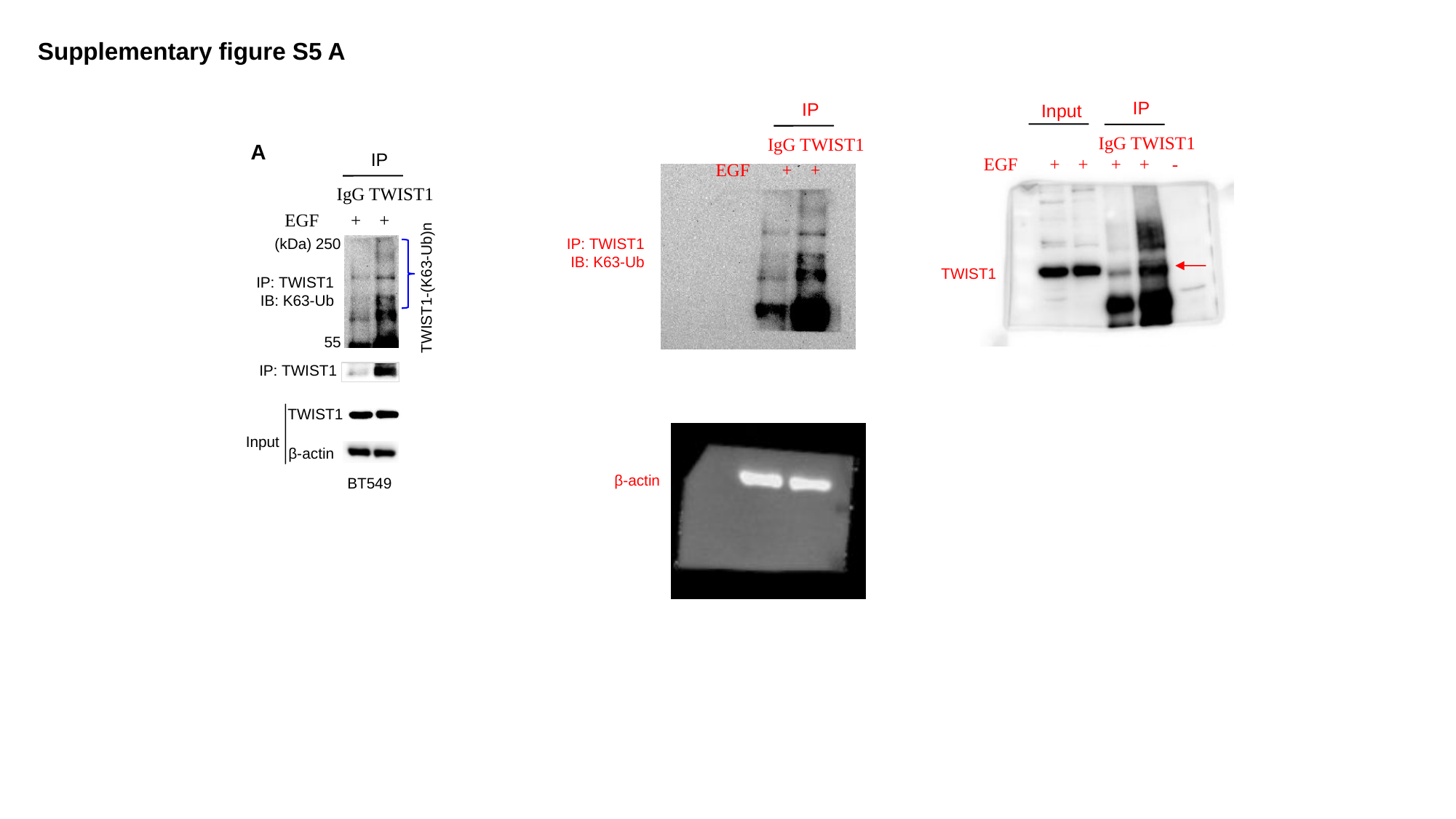

Supplementary figure S5 A
IP
IP
Input
IgG TWIST1
IgG TWIST1
A
IP
IgG TWIST1
EGF + +
(kDa) 250
55
IP: TWIST1
IB: K63-Ub
TWIST1-(K63-Ub)n
IP: TWIST1
TWIST1
Input
β-actin
BT549
EGF + + + + -
EGF + +
IP: TWIST1
IB: K63-Ub
TWIST1
β-actin

## Slide 24
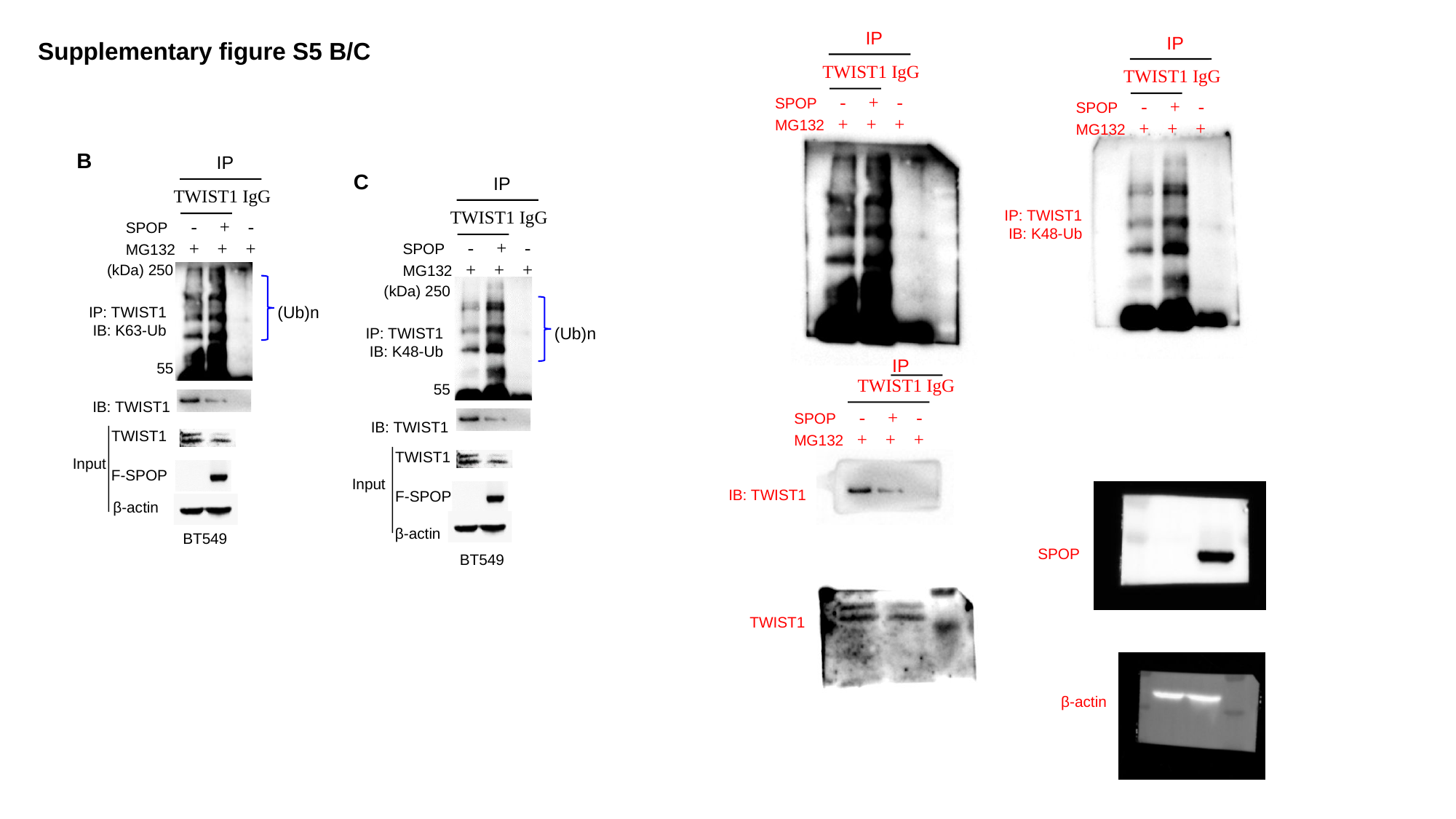

IP
IP
Supplementary figure S5 B/C
TWIST1 IgG
TWIST1 IgG
SPOP - + -
MG132 + + +
SPOP - + -
MG132 + + +
B
IP
TWIST1 IgG
SPOP - + -
MG132 + + +
(kDa) 250
55
(Ub)n
IP: TWIST1
IB: K63-Ub
IB: TWIST1
TWIST1
Input
F-SPOP
β-actin
BT549
C
IP
TWIST1 IgG
SPOP - + -
MG132 + + +
(kDa) 250
55
(Ub)n
IP: TWIST1
IB: K48-Ub
IB: TWIST1
TWIST1
Input
F-SPOP
β-actin
BT549
IP: TWIST1
IB: K48-Ub
IP
TWIST1 IgG
SPOP - + -
MG132 + + +
IB: TWIST1
SPOP
TWIST1
β-actin
